# MeCP2, a target of miR-638, facilitates gastric cancer cell proliferation through activation of the MEK1/2–ERK1/2 signaling pathway by upregulating GIT1

**DOI:** 10.1038/oncsis.2017.60

**Published:** 2017-07-31

**Authors:** L Y Zhao, D D Tong, M Xue, H L Ma, S Y Liu, J Yang, Y X Liu, B Guo, L Ni, L Y Liu, Y N Qin, L M Wang, X G Zhao, C Huang

**Affiliations:** 1 Department of Cell Biology and Genetics/Key Laboratory of Environment and Genes Related to Diseases, School of Basic Medical Sciences, Xi'an Jiaotong University Health Science Center, Xi'an, Shaanxi, China; 2 Department of Radiation Oncology, The First Affiliated Hospital of Medical College, Xi’an Jiaotong University, Xi'an, Shaanxi, China; 3 Key Laboratory of Shaanxi Province for Craniofacial Precision Medicine Research, College of Stomatology, Xi'an Jiaotong University, Xi'an, Shaanxi, China; 4 Medical College of Yan’an University, Yan'an, Shaanxi, China

## Abstract

Methyl-CpG binding protein 2 (MeCP2) is involved in the carcinogenesis and progression of multiple types of cancer. However, its precise role in gastric cancer (GC) and the relevant molecular mechanism remain unknown. In the present study, we found that miR-638 levels were lower in GC tissues and GC cell lines than in adjacent normal tissues and normal gastric epithelial cell lines, respectively. Low miR-638 levels were associated with poor tumor differentiation, tumor size and lymph node metastasis. MeCP2 expression levels were higher in GC tissues than in adjacent normal tissues. It was found that miR-638 inhibited GC cell proliferation, colony formation, G1–S transition and tumor growth, and induced cell apoptosis by directly targeting MeCP2. MeCP2 promoted GC cell proliferation, colony formation and G1–S cell-cycle transition, and suppressed apoptosis. Molecular mechanistic investigations were performed using an integrated approach with a combination of microarray analysis, chromatin immunoprecipitation sequencing and a reporter gene assay. The results showed that MeCP2 bound to the methylated CpG islands of G-protein-coupled receptor kinase-interacting protein 1 (GIT1) promoter and upregulated its expression, thereby activating the MEK1/2–ERK1/2 signaling pathway and promoting GC cell proliferation. Taken together, our study demonstrates that MeCP2, a target of miR-638, facilitates GC cell proliferation and induces cell-cycle progression through activation of the MEK1/2–ERK1/2 signaling pathway by upregulating GIT1. The findings suggest that MeCP2 plays a significant role in GC progression, and may serve as a potential target for GC therapy.

## Introduction

Gastric cancer (GC) is the second leading cause of cancer-related death worldwide, and its incidence and mortality are even higher in east Asia.^1, 2^ Although the clinical management has improved, the treatment of GC remains challenging owing to the complexity of the disease and difficulty in early diagnosis.^3^ GC is often diagnosed at an advanced stage, and the prognosis is poor. Understanding the tumorigenesis may help diagnose the disease at an earlier stage and thus increase the chance of taking action earlier and improving the prognosis. GC tumorigenesis is a multistep and multifactorial process involving diverse gene alterations, including the inactivation of tumor suppressor genes, activation of oncogenes and abnormal expression of cancer-related genes.^4, 5, 6^ It is crucial to investigate novel genes that govern the development of GC to elucidate the molecular mechanisms and develop effective therapeutic strategies.

MicroRNAs (miRNAs) are a class of endogenous, small, single-stranded non-coding RNAs, 19–25 nucleotides in length, which can bind to the 3′ untranslated regions (UTRs) of multiple target mRNAs, initiating their degradation and thereby suppressing their translation.^7, 8^ miRNAs participate in some important biological processes, such as cell proliferation, differentiation, development, stress response, inflammation, metabolism and apoptosis.^9^ Aberrantly expressed miRNAs may function as oncogenes and anti-oncogenes, contributing to carcinogenesis and cancer progression.^10^ Numerous miRNAs have been reported to be variously expressed between GC tissues and normal adjacent tissues.^11, 12^ As a member of the miRNA family, miR-638 plays a crucial role in carcinogenesis and cancer progression.^13, 14^ Our previous study has revealed that dysregulation of miR-638 is involved in GC progression.^15^ However, the molecular mechanisms underlying the role of miR-638 in GC remain unclear. We predicted and found that miR-638 can target methyl-CpG binding protein 2 (MeCP2) using bioinformatics software. MeCP2, a key epigenetic regulator, regulates chromatin organization, gene transcription and gene promoters by binding to methylated DNA.^16, 17, 18, 19^ It is a master regulator of gene expression and is a genetic cause of a variety of neurological disorders, such as Rett syndrome, and its role in neuronal systems has been well studied.^20^ Recently, MeCP2 is reported to participate in cell growth and tumorigenesis.^21, 22^ However, its role in many types of cancer, including GC, has not been well studied, and particularly the molecular mechanisms underlying its action remain unknown.

In the present study, we characterized the role and mechanisms of miR-638-mediated MeCP2 in GC proliferation. We found that the expression of MeCP2 was remarkably upregulated in GC tissues and the expression of miR-638 was downregulated. miR-638-mediated MeCP2 promoted GC cell proliferation. Further molecular mechanistic investigations revealed that MeCP2 promoted GC cell growth through activation of the mitogen/extracellular signal/regulated kinase1/2–extracellular regulated protein kinase1/2 (MEK1/2–ERK1/2) signaling pathway by upregulating G-protein-coupled receptor kinase-interacting protein 1 (GIT1).

## Results

### miRNA-638 is specifically downregulated in GC tissues

**Figure 1:**
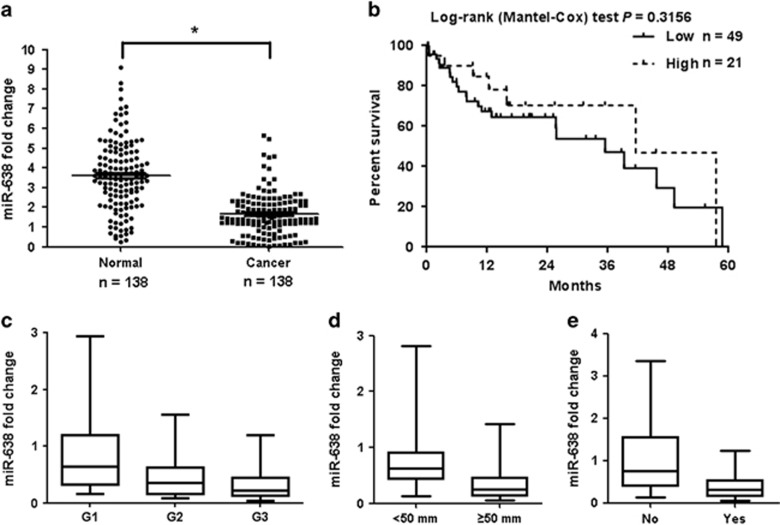
miR-638 expression decreases in human GC. (**a**) miR-638 expression in GC tissues and normal tissues. *P*=0.003. (**b**) Kaplan–Meier analysis of the effect of miR-638 expression on the overall survival of 70 GC patients. (**c**) miR-638 expression in different histological grades of GC samples. *P*=0.009. (**d**) miR-638 expression in tumor samples with different sizes. *P*=0.015. (**e**) miR-638 expression in tumor samples with different lymph node metastasis states. *P*=0.026. For (**c**–**e**), whiskers represent the 5th and 95th percentiles.

To analyze the role of miR-638 in GC progression, miR-638 expression was examined in clinical samples (138 GC tissue specimens and 138 adjacent normal (non-tumor) gastric tissues) and tumor cell lines. As shown in Figure 1a, miR-638 levels in the GC tissues were significantly lower than those in the normal tissues (*P*=0.003). Further investigation revealed that 84.1% of the level of miR-638 expression in cancer tissue exhibited lower expression levels than that in its adjacent cancer tissue. However, the results did not show a correlation between miR-638 expression and the overall survival of GC patients (*P*>0.05; Figure 1b). Figures 1c–e and Supplementary Table S1 present the correlation between miR-638 levels and clinicopathologic characteristics of GC patients. Low miR-638 expression was associated with poor tumor histology (well: 73.7% moderate: 89.5% poor: 93.0%), tumor size (tumor size <50 mm: 77.8% tumor size ⩾50 mm: 89.3%) and lymph node metastasis (Yes: 93.8% No: 62.8%). Additionally, miR-638 expression was significantly lower in GC cell lines (BGC-823, AGS, SGC-7901 and MKN-45) than in a normal human gastric epithelial cell line (GES-1) (*P*=0.003; Supplementary Figure S1a). These data indicated that miR-638 functioned as a potential tumor suppressor in GC.

### MeCP2 is significantly upregulated in GC tissues and cell lines

**Figure 2:**
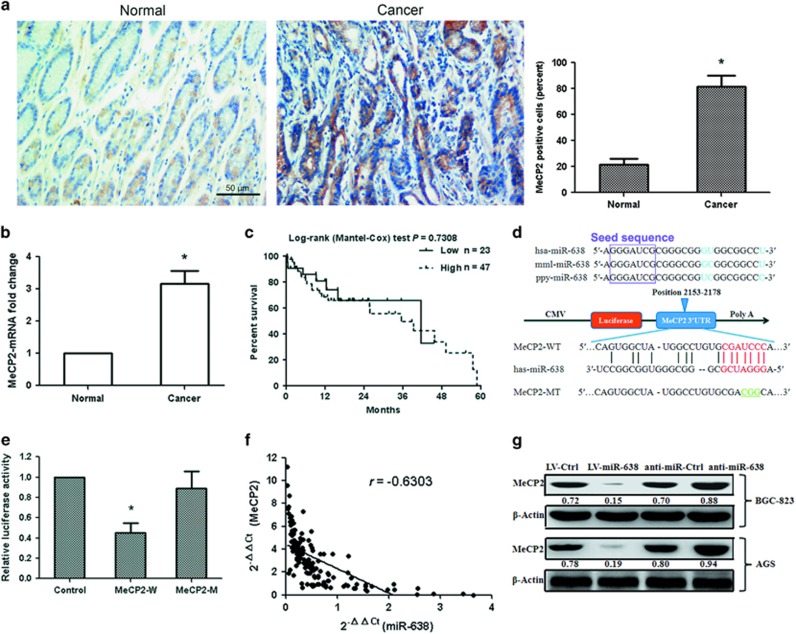
miR-638 downregulates MeCP2 expression by targeting its 3′-UTR. (**a**) MeCP2 protein levels in tumor tissues and normal tissues were measured by immunohistochemical staining. *n*=76, *P<0.01. (**b**) MeCP2 mRNA expression was determined in tumor tissues and normal tissues. *n*=138, *P<0.01. (**c**) Kaplan–Meier analysis of the effect of MeCP2 mRNA expression on the overall survival of 70 GC patients. (**d**) Conserved miR-638 seed region sequence in the 3′-UTR of MeCP2 in various species. (**e**) The luciferase reporter plasmid containing wild-type (WT) or mutant-type (MT) MeCP2 3′-UTR was cotransfected with miR-638. *P<0.01. (**f**) Expression levels of miR-638 and MeCP2 were inversely correlated. The 2^−ΔΔCt^ values of miR-638 and MeCP2 mRNA were subjected to a Pearson correlation analysis (*r*=−0.6303, *n*=138, *P*<0.001). (**g**) Expression of MeCP2 protein was analyzed by western blotting, using β-actin as the internal control.

MeCP2, SP2 and PLD1 were identified as candidate targets of miR-638 by bioinformatics analysis (RegRNA, PicTar and TargetScan). The relationship between miR-638 and MeCP2 is the focus in the present study. To evaluate the potential clinical significance of MeCP2, we measured the mRNA and protein expressions of MeCP2 using quantitative real-time PCR (qRT–PCR) and immunohistochemical staining, in 138 GC tissue samples and 138 adjacent normal gastric tissue samples. The results showed that MeCP2 protein and mRNA levels were remarkably higher in GC tissues (*P*=0.002; Figures 2a and b). However, the mRNA expression is not related to the overall survival of GC patients (*P*>0.05; Figure 2c). In addition, MeCP2 expression in GC cell lines increased significantly (BGC-823 and AGS) (*P*=0.003; Supplementary Figure S1b). These results suggested that upregulated MeCP2 expression was involved in the progression of human GC.

### miRNA-638 directly targets MeCP2

To assess the relationship between miR-638 and MeCP2, we constructed 3′-UTR fragments of MeCP2, in which wild-type (WT) and mutant-type (MT) miR-638-binding sites were subcloned into the pmiRGLO dual-luciferase reporter vector (Figure 2d). We then cotransfected HEK293T cells with WT 3′-UTR or MT 3′-UTR constructs and pre-miR-638. The results showed that pre-miR-638 induced a remarkable reduction in luciferase activity of the WT 3′-UTR (MeCP2) reporter construct (*P*=0.002); however, miR-638 failed to inhibit the luciferase activity of the reporter construct containing the MT binding site, suggesting that miR-638 directly binds to the 3′-UTR of MeCP2 (Figure 2e). A significant inverse correlation was observed between the miR-638 expression and MeCP2 mRNA level in the GC specimens (*n*=138, *r*=−0.6303, *P*=0.002, Pearson’s correlation; Figure 2f), suggesting that the target effect of miR-638 on MeCP2 is clinically relevant in GC. The ability of miR-638 to regulate MeCP2 was evaluated using qRT–PCR, and the results showed that the miR-638 expression in cells transfected with LV-miR-638 vector was higher than that in cells transfected with LV-Ctrl (*P*=0.001; Supplementary Figure S1c). Overexpression of miR-638 significantly reduced the mRNA (*P*=0.004) and protein levels of MeCP2, whereas inhibition of miR-638 led to the upregulation of MeCP2 at mRNA (*P*=0.005) and protein levels (Supplementary Figure S1d and Figure 2g). The data here demonstrated that miR-638 post-transcriptionally regulated MeCP2 by directly targeting its 3′-UTR in GC cells.

### miRNA-638 suppresses GC cell growth *in vitro* and *in vivo*

**Figure 3:**
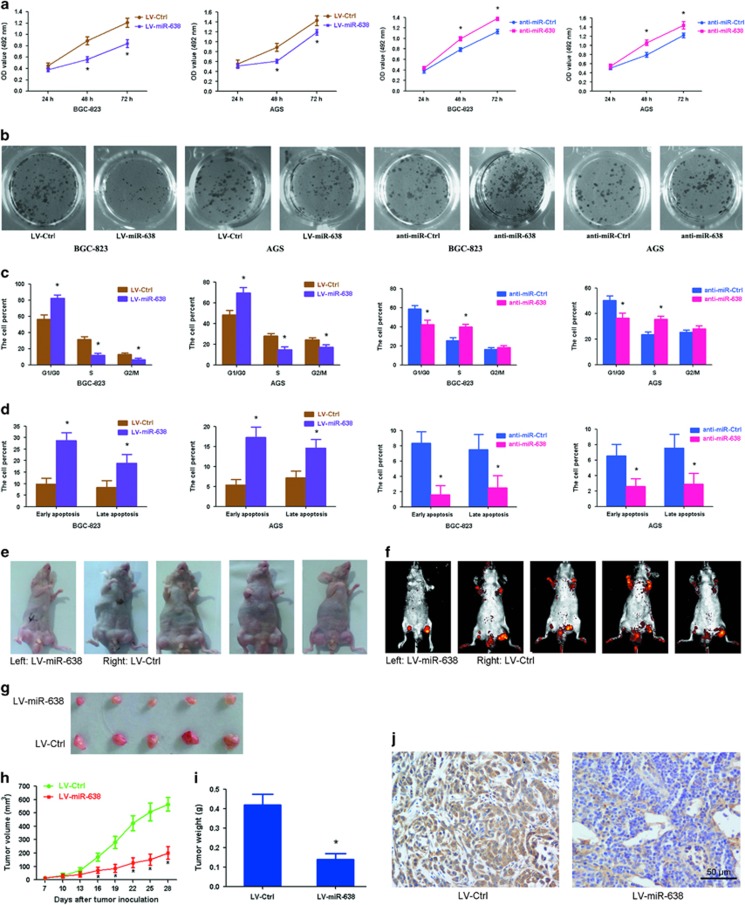
miR-638 inhibits GC cell proliferation *in vitro* and *in vivo*. (**a**) Cell proliferation was examined by MTT assay at 24, 48 and 72 h after transfection with LV-miR-638 or anti-miR-638. *P<0.01. (**b**) Cell colonies were analyzed by colony formation assay 12 days after transfection. (**c**) Detection of cell cycle by flow cytometry analysis was visualized using propidium iodide staining. Histograms showed the percentage of cells in the G1/G0, S and G2/M phases. *P<0.01. (**d**) Detection of cell apoptosis by flow cytometry analysis was visualized using Annexin-V/propidium iodide staining. The data showed the percentage of early- and late-apoptotic cells. *P<0.01. (**e**) Gross morphology of tumors injected with either LV-miR-638 or LV-Ctrl cells after 28 days. (**f**) Small animal imaging analysis was used to assess tumor volume *in situ* at day 28 during tumor development. (**g**) Morphology of excised tumors from nude mice. (**h**) Growth curves of tumor volume were generated every 3 days for 21 days. *P<0.01. (**i**) Tumors were weighed at day 28 after initial injection. *P<0.01, *n*=5. (**j**) Immunohistochemical staining of MeCP2 in tumor tissues from LV-Ctrl- and LV-miR-638-injected mice.

In order to confirm whether the effects of miR-638 on proliferation observed in GC cells and clinical samples were related to tumor growth, BGC-823 and AGS cells were transfected separately with LV-miR-638 and anti-miR-638 (lentiviral vectors). Results from (3-(4,5-dimethylthiazol-2-yl)-2,5-diphenyltetrazoliumbromide) (MTT) assays showed that proliferation was inhibited in LV-miR-638-transfected cells, but was promoted in anti-miR-638-transfected cells (Figure 3a). Similar results were observed for colony formation. Pre-miR-638 suppressed colony formation while anti-miR-638 promoted colony formation in these cells (Figure 3b and Supplementary Figures S1e and f). Cell-cycle status of each cell line was examined using a flow cytometer 2 days after treatment. It was found that miR-638 overexpression triggered the accumulation of cells at the G1/G0 phase and decreased the proportion of cells in the S and G2/M phases (*P*=0.006); however, opposite results were observed in cells transfected with anti-miR-638 (*P*=0.039; Figure 3c). In addition, evaluation of cell apoptosis by Annexin-V/propidium iodide staining identified that the proportion of early-apoptotic and late-apoptotic cells increased significantly when miR-638 was overexpressed (*P*=0.007), and decreased remarkably when anti-miR-638 was overexpressed (*P*=0.021; Figure 3d).

In an attempt to further investigate the effect of miR-638 on GC cell growth *in vivo*, LV-miR-638-infected and LV-Ctrl-infected BGC-823 cells were injected subcutaneously into both posterior flanks of the same nude mouse (left and right) and tumor growth was observed over a 4-week period following injection. The results showed that tumor growth was significantly suppressed by LV-miR-638 as compared with that seen in the LV-Ctrl group (Figures 3e–g and Supplementary Figure S2a). This trend was confirmed by the weights and sizes of tumors excised from the mice. At day 28, the average volume of tumors injected with LV-Ctrl was approximately 2.79-fold higher than that of tumors injected with LV-miR-638 (*n*=5, *P*=0.003; Figure 3h). The average weight of tumors was 0.42 g in the LV-Ctrl group and 0.14 g in the LV-miR-638 group (*P*=0.001; Figure 3i). Furthermore, the expression of miR-638 in LV-miR-638 tumors was approximately 31.2-fold higher than that in LV-Ctrl tumors (Supplementary Figure S2b). The mRNA and protein expressions of MeCP2 were significantly downregulated in the LV-miR-638 group as compared with that in the LV-Ctrl group (*P*=0.002; Figure 3j and Supplementary Figures S2c–e). Based on these findings, we concluded that miR-638 suppresses GC cell proliferation by downregulating MeCP2.

### MeCP2 facilitates GC cell growth *in vitro*

**Figure 4:**
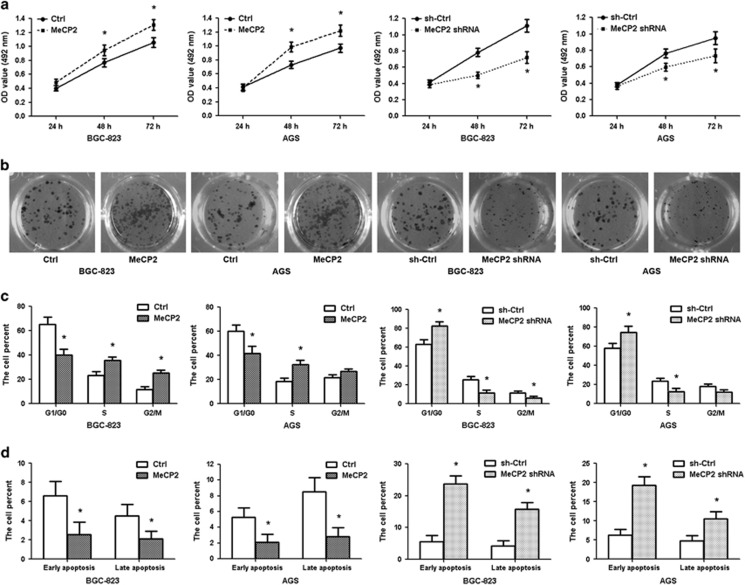
MeCP2 promotes GC cell growth. (**a**) MTT assay showed that MeCP2 promoted GC cell proliferation after transfection. *P<0.01. (**b**) Cell colonies were examined 12 days after transfection. (**c**) Histograms showed the percentage of cells in the G1/G0, S and G2/M phases after transfection. *P<0.01. (**d**) Percentages of early- and late-apoptotic cells. *P<0.01.

Lentivirus-mediated shRNA was used to silence endogenous MeCP2 expression in BGC-823 and AGS cells, and an overexpression vector harboring the MeCP2 gene was constructed. The mRNA and protein levels of MeCP2 confirmed that the expression of this gene is specifically knocked down by shRNA (*P*=0.004) and upregulated by the overexpression vector (*P*=0.001; Supplementary Figures S3a and b). As shown in Figures 4a–d and Supplementary Figures S3c and d, silencing MeCP2 resulted in the suppression of cell proliferation and colony formation, induction of G1/G0 cell-cycle arrest and promotion of apoptosis. These effects were in line with those of miR-638 overexpression. In contrast, MeCP2 overexpression promoted cell growth and colony formation, induced G1–S phase transition and inhibited apoptosis. These data suggested that MeCP2 drove G1–S transition in the cell cycle and consequently promoted the proliferation of GC cells.

### Overexpression of MeCP2 eliminated the effects of miR-638 on GC cells

To further demonstrate that miR-638 suppressed tumor by functioning through MeCP2, an overexpression vector for MeCP2 was cotransfected with LV-miR-638 into BGC-823 or AGS cells. Overexpression of MeCP2 rescued the MeCP2 level reduced by miR-638 (Supplementary Figures S4a and b). It also counterbalanced the tumor suppressor effect of miR-638 in GC cell proliferation (Supplementary Figures S4c and d). Cell-cycle assay showed that MeCP2 was able to re-enter the S and G2/M phases in GC cells compared with the miR-638 group (Supplementary Figure S4e). Meanwhile, cotransfected with miR-638 and MeCP2 could inhibit apoptosis after the cotrasfection, as compared with that in cells transfected with only miR-638 (Supplementary Figure S4f). These results further demonstrated that miR-638 played a tumor suppressor role by directly targeting MeCP2.

### MeCP2 promotes the expression of GIT1 by binding its promoter

**Figure 5:**
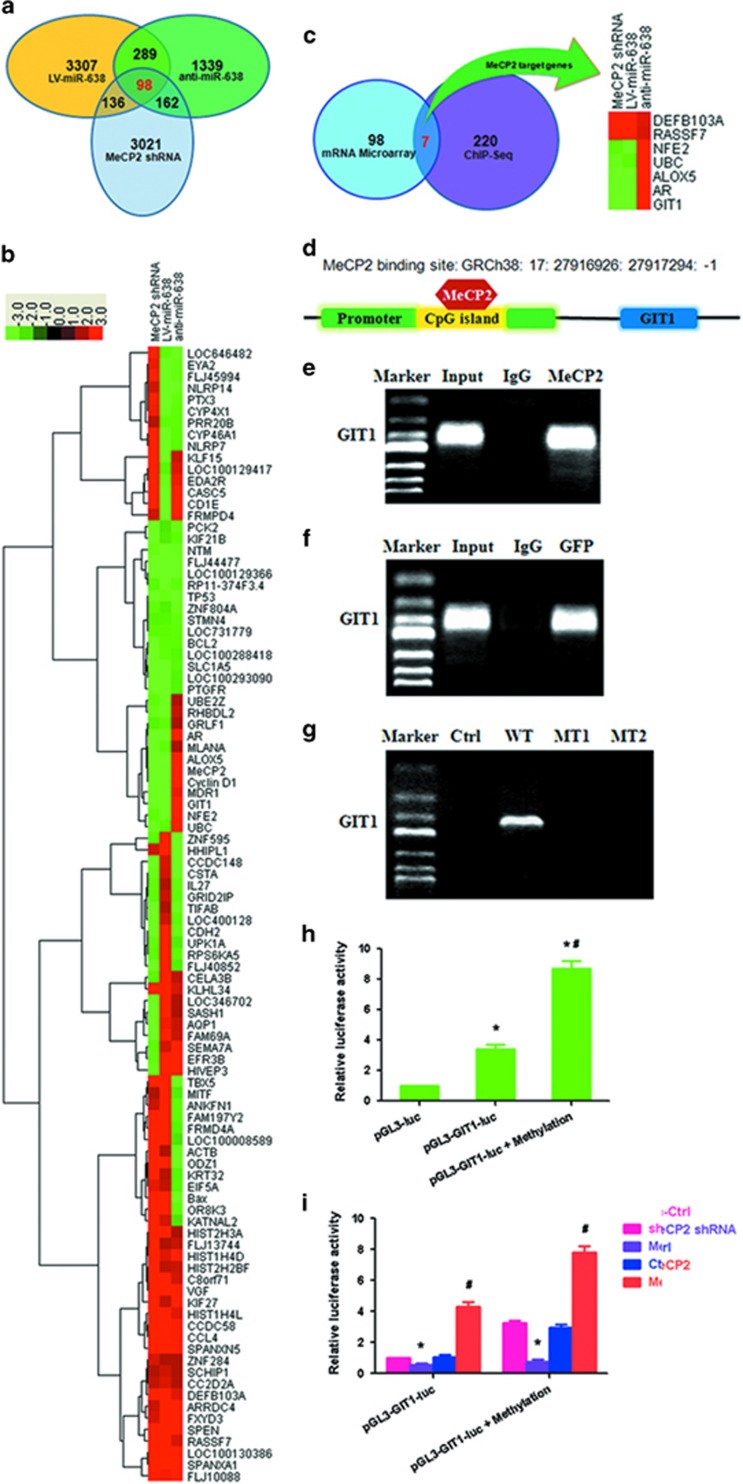
MeCP2 binds to the promoter regions of GIT1 in GC cells. (**a**) Overlapping genes (*n*=98) after transfection with MeCP2 shRNA, LV-miR-638 or anti-miR-638. (**b**) Heat map of 98 overlapping genes with fold change⩾2.0 after transfection with MeCP2 shRNA, LV-miR-638 or anti-miR-638. (**c**) Overlapping genes (*n*=98) after transfection with MeCP2 shRNA, LV-miR-638 or anti-miR-638 compared with 220 genes corresponding to ChIP-Seq peaks located in the promoter. (**d**) MeCP2 binding site in the promoter of GIT1. (**e**) ChIP–RT–PCR of GIT1 performed with anti-MeCP2 antibody. (**f**) ChIP–RT–PCR of GIT1 performed with anti-GFP antibody after transfection with GFP-MeCP2 plasmid. (**g**) ChIP–RT–PCR of GIT1 performed with anti-GFP antibody after transfection with Ctrl (GFP), WT (GFP-MeCP2), MT1 (GFP-Mutation type 1) or MT2 (GFP-Mutation type 2) plasmids. (**h**) BGC-823 cells were transfected with pGL3-GIT1-luc (target sequences of promoter regions of GIT1) or pGL3-GIT1-luc+methylation; luciferase activity was determined at 48 h post-transfection. *Renilla* luciferase served as the internal control. *P<0.01 compared with pGL3-luc. ^#^*P*<0.01 compared with pGL3-GIT1-luc. (**i**) BGC-823 cells were treated with pGL3-GIT1-luc, methylation, MeCP2 shRNA or MeCP2 overexpression vector; luciferase activity was determined. *P<0.01 compared with sh-Ctrl. ^#^*P*<0.01 compared with Ctrl.

To identify genes regulated by miR-638-mediated MeCP2, global gene expression was profiled using microarray. The results showed that expression of 3021, 3307 and 1339 genes was significantly altered following transfection with MeCP2 shRNA, LV-miR-638 and anti-miR-638, respectively (fold change⩾2.0; Figure 5a). Bioinformatics analysis identified 98 overlapping genes showing changes in expression after transfection, among which 24 were regulated by miR-638-mediated MeCP2, including UBE2Z, RHBDL2, GRLF1, AR, MLANA, ALOX5, Cyclin D1, MDR1, GIT1, NFE2, and UBC that were upregulated, and TBX5, MITF, ANKFN1, FAM197Y2, FRMD4A, LOC100008589, ACTB, ODZ1, KRT32, EIF5A, Bax, OR8K3 and KATNAL2 that were downregulated (Figure 5b). The 98 genes were then subjected to GO analysis using the KEGG database (http://www.genome.jp/kegg/) to associate differentially expressed mRNAs with GO categories. The top 10 GO functions regulated in the biological processes, cellular components, molecular functions and pathway genes are shown in Supplementary Figures S5a–d. The pathways included isopeptide bond, ubl conjugation, methylated amino acid, protein N-terminus binding, transcription activator activity, BH domain binding, BH3 domain binding, acetyllysine, histone core and nucleosome core.

Chromatin immunoprecipitation sequencing (ChIP-Seq) assay was performed to identify direct target genes of MeCP2 in GC cells. MeCP2 protein was observed to localize around transcription start sites. The analysis results showed that 220 of the peaks were located in the promoter regions of genes (enrichment folds⩾2). The 98 overlapping genes regulated by miR-638-mediated MeCP2 were compared with those bound by MeCP2. As a result, seven direct target genes were initially identified, of which five were activated and two were repressed by MeCP2. The direct target genes were DEFB103A, RASSF7, NFE2, UBC, ALOX5, AR and GIT1 (Figure 5c). The sites and enrichment of MeCP2 binding these seven genes are shown in Supplementary Table S2. Next, we predicted a CpG site at MeCP2 binding sites via using MethPrimer. It was found that MeCP2 sites in the promoter regions of GIT1 contained a CpG site (Figure 5d). ChIP–RT–PCR verified that MeCP2 directly bound to the promoters of GIT1 (Figure 5e). We constructed WT and MT GFP-MeCP2 plasmids. After transfection into BGC-823 cells, we performed ChIP–RT–PCR with an anti-GFP antibody. We found that exogenous MeCP2 also bound to the CpG island of promoter regions of GIT1 (Figure 5f). GFP plasmid (Ctrl), GFP-MT1 and GFP-MT2 did not bind to the CpG island of GIT1, but GFP-WT did (Figure 5g). A promoter reporter assay was performed to detect whether MeCP2 bound to the CpG island of GIT1. The binding sequence of GIT1 from ChIP-Seq were subcloned upstream of the luciferase gene in the pGL3 reporter vector (Supplementary Table S2). Luciferase activity was examined at 48 h post-transfection in BGC-823 cells. The results showed that luciferase activity remarkably increased in the pGL3-GIT1-luc and pGL3-GIT1-luc+methylation groups as compared with that of the pGL3 group (Figure 5h). When pGL3-GIT1-luc was transfected, luciferase activity decreased in the MeCP2 shRNA group as compared with the sh-Ctrl group, and increased in the MeCP2 overexpression group as compared with the Ctrl group. Transfection with pGL3-GIT1-luc+methylation vector led to similar results, and the activity was higher than those in the pGL3-GIT1-luc groups (Figure 5i). These results confirmed MeCP2 as a regulator of GIT1 in GC cells.

**Figure 6:**
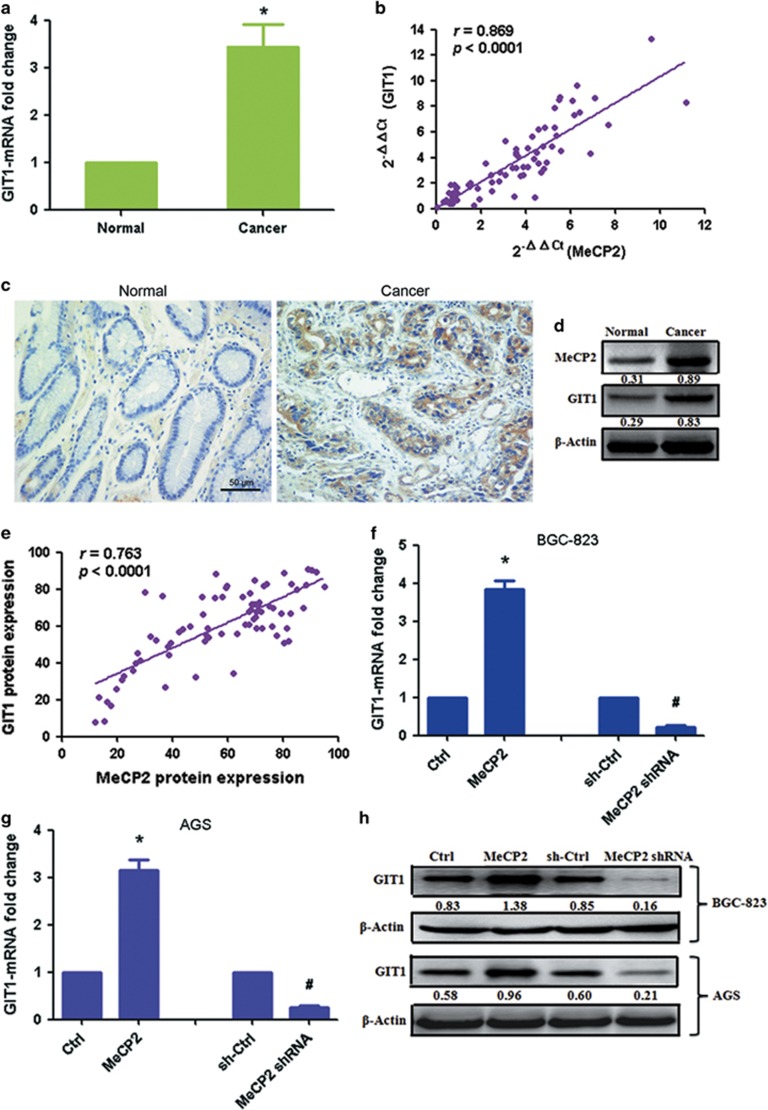
MeCP2 facilitates the expression of GIT1 in GC cells. (**a**) GIT1 mRNA expression in GC tissues versus normal tissues. *P<0.01. (**b**) MeCP2 and GIT1 expressions showed a positive correlation. The 2^−ΔΔCt^ values of MeCP2 and GIT1 mRNA were subjected to a Pearson correlation analysis (*n*=76, *r*=0.869, *P*<0.0001). (**c**) Protein levels of GIT1 in tumor tissues and normal tissues were measured by immunohistochemical staining. (**d**) Protein expression levels of GIT1 and MeCP2 were measured by western blotting. (**e**) MeCP2 and GIT1 protein expressions showed a positive correlation (*n*=76, *r*=0.763, *P*<0.0001). (**f**) GIT1 mRNA expression in BGC-823 cells transfected with MeCP2 shRNA or MeCP2 overexpression vector. *P<0.01 compared with Ctrl, ^#^*P*<0.01 compared with sh-Ctrl, *n*=3. (**g**) GIT1 mRNA expression in AGS cells after transfection. *P<0.01 compared with Ctrl, ^#^*P*<0.01 compared with sh-Ctrl, *n*=3. (**h**) GIT1 protein expression after transfection with MeCP2 shRNA or MeCP2 overexpression vector.

To further validate this finding, we dectected the expression of GIT1 in GC tissues. GIT1 mRNA was found significantly upregulated in GC tissues compared with in normal tissues (Figure 6a). MeCP2 mRNA expression was significantly positively correlated with GIT1 mRNA levels in GC specimens (*n*=76, *r*=0.869, *P*<0.0001, Pearson’s correlation; Figure 6b). GIT1 protein expression also increased in GC tissues (Figures 6c and d). High GIT1 protein expression was associated with poor tumor histology (well: 63.9% (23/36); moderate: 94.4% (17/18); poor: 90.9% (20/22)) (Supplementary Table S3). However, the expression was not associated with age, gender, tumor size, lymph node metastasis, lymphatic invasion, venous invasion, T stage and TNM stage. There was also a positive correlation between MeCP2 and GIT1 protein expressions (*n*=76, *r*=0.763, *P*<0.0001; Figure 6e). In addition, analysis of GIT1 mRNA levels after transfection with MeCP2 overexpression vector or MeCP2 shRNA revealed that GIT1 mRNA expression in the GC cells was increased by MeCP2 overexpression and reduced by MeCP2 shRNA (Figures 6f and g). Similarly, GIT1 protein levels increased after transfection with MeCP2 overexpression vector and decreased after transfection with MeCP2 shRNA (Figure 6h). These data demonstrated that MeCP2 activated the expression of GIT1 in GC cells by binding to the CpG site at the promoter.

### MeCP2 activates the MEK1/2–ERK1/2 signaling pathway via upregulation of GIT1 leading to increased cell proliferation

**Figure 7:**
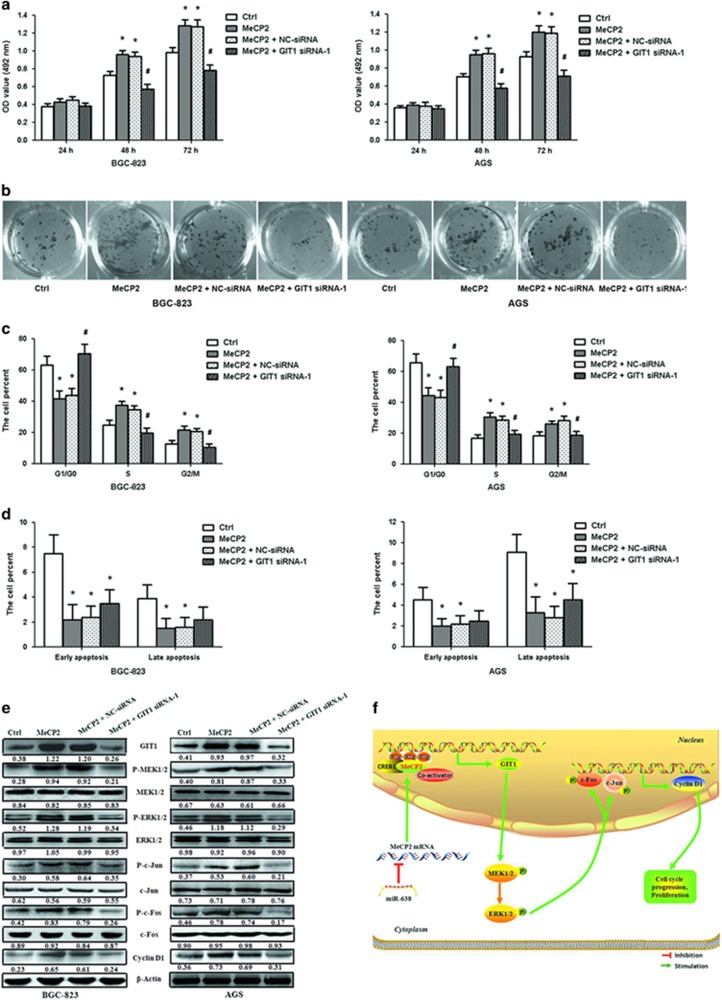
MeCP2 promotes GC cell proliferation by regulating the GIT1–MEK1/2–ERK1/2 signaling pathway. (**a**) MTT assay showed GC cell proliferation in BGC-823 and AGS cells after cotransfection with MeCP2 overexpression vector and GIT1 siRNA-1. (**b**) Cell colonies were examined 12 days after cotransfection. (**c**) Percentage of cells in the G1/G0, S and G2/M phases after cotransfection. (**d**) Percentages of early- and late-apoptotic cells after cotransfection. (**e**) GIT1–MEK1/2–ERK1/2 signaling pathway was detected after cotransfection. *P<0.01 compared with Ctrl cells, ^#^*P*<0.01 compared with cells transfected with MeCP2 overexpression vector alone. *n*=3. (**f**) Proposed model for the effects of miR-638-mediated MeCP2 on GC proliferation via regulation of the GIT1–MEK1/2–ERK1/2 signaling pathway. miR-638 expression downregulates the expression of MeCP2. MeCP2 facilitates GC cell proliferation by binding to a methylated CpG site in the GIT1 promoter regions and promoting GIT1 expression, which further activates the MEK1/2–ERK1/2 signaling pathway.

To further confirm that MeCP2 promoted GC cell proliferation by facilitating GIT1 expression, we synthesized GIT1 siRNA-1 and siRNA-2 for efficient silencing GIT1 (Supplementary Figures S6a and b). GIT1 siRNA-1 was cotransfected with MeCP2 overexpression vector into GC cells. Cell viability and colony formation assays showed that knockdown of GIT1 abrogated the effect of MeCP2 overexpression on cell proliferation (Figures 7a and b and Supplementary Figure S6c). Cell-cycle analysis also revealed that cells cotransfected with MeCP2 overexpression vector and GIT1 siRNA-1 were better able to induce G1/G0 cell-cycle arrest as compared with cells transfected with MeCP2 overexpression vector alone (Figure 7c). Evaluation of apoptotic status revealed no significant difference between these two groups of cells (Figure 7d). Previous studies have reported that GIT1 activates the MEK1/2–ERK1/2 signaling pathway.^23^ Therefore, we adopted MEK1/2 inhibitor to examine the role of MEK1/2–ERK1/2 signaling pathway in GC progression. BGC-823 and AGS cells were treated with MeCP2 overexpression vector and MEK1/2 inhibitor (U0126). Cell viability and colony formation assays showed that U0126 eliminated the effect of MeCP2 overexpression on cell proliferation (Supplementary Figures S7a and b). Cell-cycle analysis showed that MeCP2+U0126 induced G1/G0 cell-cycle arrest as compared with MeCP2 overexpression (Supplementary Figure 7c). No significant differences were observed in the distribution of apoptosis between the MeCP2+U0126 and MeCP2 overexpression groups (Supplementary Figure 7d).

The downstream regulators involved in the promotion of proliferation by MeCP2 were examined by western blot analyses. Upregulation of MeCP2 increased GIT1, P-MEK1/2, P-ERK1/2, P-c-Jun, P-c-Fos and cyclin D1 protein expression levels. Compared with those in cells transfected with MeCP2 overexpression vector alone, the protein levels of these downstream regulators were found to be significantly downregulated in the cotransfected cells. No significant differences were observed in the expression of MEK1/2, ERK1/2, c-Jun and c-Fos (Figure 7e). These results further demonstrated that MeCP2 promoted GC cell proliferation by upregulating the expression of GIT1, which in turn activated the MEK1/2–ERK1/2 signaling pathway.

## Discussion

In the last decade, accumulating evidence suggests that miRNAs play important roles in controlling cancer-related cell functions, such as proliferation, survival, differentiation, metastasis, invasion and apoptosis.^24, 25, 26^ Aberrantly expressed miRNAs are found to be involved in gastric carcinogenesis and progression.^27, 28^ Identifying miRNAs and elucidating their precise biological roles in GC will aid the search for new targets for diagnosis and therapy of the disease. miRNA-638 has been reported to regulate the progression of several human cancers, including colorectal carcinoma, lung carcinoma, melanoma, breast cancer and leukemia.^14, 29^ Our previous study and Zhang’s findings show that miR-638 expression decreases in GC tissues.^15, 30^ In this study, we further verify that miR-638 expression is frequently downregulated in both GC tissues and cell lines. The clinicopathological significance of miR-638 expression was also analyzed. We found that low miR-638 levels are significantly associated with poor tumor differentiation, tumor size and lymph node metastasis. No significant correlation is identified between miR-638 expression and other clinicopathological characteristics. Our *in vitro* experiment shows that miR-638 suppresses GC cell proliferation by blocking G1–S transition and induces apoptosis, and the *in vivo* experiment demonstrates that miR-638 inhibits tumor growth. To sum up, miR-638 acts as an anti-oncogene in GC, and shows potential for being a diagnostic marker and therapeutic target for GC.

The role of MeCP2 in malignancy has not been well studied. Emerging evidence suggests MeCP2 as a key oncogene in tumorigenesis and development. Neupane *et al.*^31^ identified MeCP2 as a frequently amplified oncogene in several cancer types, including breast cancer, cervical cancer, lung cancer and uterine cancer, and they reported the ability of MeCP2 to drive breast cancer development. Another study reported a higher MeCP2 expression in neoplastic breast tissues than in non-neoplastic tissues.^32^ Our previous study observed remarkable upregulation of MeCP2 expression in human hepatocellular carcinoma tissues and the ability of MeCP2 to promote cell proliferation.^22^ The present study again demonstrates that MeCP2 promotes GC cell proliferation, and the promotion is achieved by facilitating G1–S transition and inhibiting apoptosis. Our previous study identified that MeCP2 shRNA suppressed GC tumor growth.^33^ The present study goes a step forward, demonstrating that miR-638 suppresses MeCP2 expression by binding directly to the 3′-UTR of MeCP2, and there is an inverse correlation between miR-638 and MeCP2 expression in GC tissues. miR-638 overexpression results in the inhibition of GC cell proliferation via miR-638-mediated downregulation of MeCP2 expression. In brief, miR-638 suppresses GC cell proliferation and induces apoptosis by directly targeting MeCP2.

As a master regulator of gene expression on the one hand, MeCP2 acts as a transcriptional repressor by binding methylated CpG dinucleotides and recruiting corepressors, such as HDAC and Sin3A, to the promoter regions to suppress the expression of a variety of genes, such as BDNF and Cdkl5.^34, 35, 36^ On the other hand, MeCP2 functions as a transcriptional activator by binding methylated CpG islands and recruiting activators such as CREB1.^18, 37, 38, 39, 40, 41^ To reveal the molecular mechanisms of miR-638-mediated MeCP2 regulation in GC, we performed microarray and ChIP-Seq analyses, through which we located GIT1 as a target gene of MeCP2. Our results confirm that MeCP2 binds to the methylated CpG islands in the promoter regions of GIT1, resulting in upregulation of GIT1 expression.

GIT1 has multiple domains, including Spa2 homology, ARFGAP, three ankyrin repeat, paxillin-binding and coiled-coil domains, and it is known to interact with diverse molecules.^42, 43^ GIT1 plays a functional role in cell migration/invasion, lamellipodia formation, focal adhesion formation, endocytosis, angiogenesis, centrosome dynamics and cell growth.^44, 45, 46, 47, 48^ Studies have revealed the involvement of GIT1 in breast cancer, oral squamous cell carcinoma and lung cancer metastasis.^49, 50^ It is reported that the expression of GIT1 is upregulated in oral, cervical, breast, liver and colon cancers.^48, 49, 50, 51^ For example, Yoo *et al.*^49^ have found a correlation of GIT1 expression with tumor grade in cervical cancer, and Wang *et al.*^50^ have observed that higher GIT1 expression is associated with advanced grades and lymph node metastasis of oral squamous cell carcinoma. Recently, Peng *et al.*^48^ have shown that GIT1 can interact with methionine adenosyltransferase 2B variants and form a scaffold, which recruits and activates MEK1/2 and ERK1/2 to promote cell growth and tumorigenesis in human liver and colon cancer. Li *et al.*^52^ have also found that GIT1 facilitates cell proliferation in non-small cell lung cancer. In this study, we have observed upregulated GIT1 expression in GC tissues. Gene knockdown and rescue studies suggest that MeCP2 promotes GC cell proliferation through activation of the MEK1/2–ERK1/2 signaling pathway by upregulation of GIT1 (Figure 7f). During the course of this study, Neupane *et al.* reported that MeCP2 activates MAPK signaling in breast cancer, cervical cancer and lung cancer.^31^ Different from their work, our study has characterized the role of MeCP2 in GC, a previously unstudied area, and most importantly, we have revealed for the first time that miR-638-mediated MeCP2 activates the MEK1/2–ERK1/2 signaling pathway by upregulating GIT1 expression in GC.

In summary, the present study demonstrates that MeCP2, a target of miR-638, functions as an oncogene that facilitates GC cell proliferation and induces cell-cycle progression through activation of the MEK1/2–ERK1/2 signaling pathway by upregulating GIT1. Our findings suggest that MeCP2 plays a significant role in GC progression, and may serve as a potential novel target for GC therapy.

## Materials and methods

### Human GC tissue specimens and cell lines

Tumor tissues and adjacent non-tumor tissues were randomly collected from 138 GC patients treated between April 2012 and October 2014 at the Department of Oncology Surgery, the First Affiliated Hospital of Medical College, Xi'an Jiaotong University, PR China. None of the patients reported to have been pretreated with chemotherapy or radiotherapy. By reviewing the patients’ pathology records, we obtained their clinicopathological such as their age and gender, histology data, lymph node metastasis status, lymphatic and venous invasion status, tumor size, T stage and pTNM stage. Tumor stage was defined based on the criteria of the American Joint Committee on Cancer (AJCC). The study was approved by the Ethical Committee of Xi'an Jiaotong University. Informed consent was obtained from all the patients before samples were collected. Human GC cell lines, including BGC-823, AGS, MKN-45, SGC-7901 and GES-1, were obtained from the Cell Bank (Shanghai Genechem Co., Ltd, Shanghai, China). The cell lines have been authenticated and tested by the Cell Bank. For verification, we performed mycoplasma tests in our laboratory, and the cell behavior and morphology were proved consistent with the descriptions in the Cell Bank. Cells (1 × 10^5^ cells/ml) were cultured in RPMI-1640 (Gibco BRL, Grand Island, NY, USA) supplemented with 10% fetal bovine serum (Gibco) at 37 °C.

### Animals

Five-week-old male BALB/c nude mice, provided by the Central Animal Laboratory in Xi'an Jiaotong University Health Science Center, were bred under specific pathogen-free conditions. The investigator was blinded to group allocation during the experiments. All animal experiments were approved by the Institutional Animal Care and Use Committee of Xi’an Jiaotong University and were performed in accordance with the institutional guidelines for the use of laboratory animals.

### RNA extraction and qRT–PCR

Total RNA was extracted from frozen tissues and cell lines using TRIzol Reagent (Invitrogen, Carlsbad, CA, USA). qRT–PCR was performed using the SYBR Green PCR kit (Takara Biotechnology, Dalian, China); the primer sequences are listed in Supplementary Table S4. All qRT–PCR reactions were performed in triplicates for each sample with an FTC-3000 P qRT-PCR Detection System (Funglyn Biotech, Scarborough, CA, USA). β-Actin and U6 were used as controls for mRNA and miRNA, respectively.

### Immunohistochemistry

Formaldehyde-fixed, paraffin-embedded tissue samples, including specimens from the GC patients and xenograft tumor tissues, were sectioned at a thickness of 4 μm. The sections were deparaffinized with xylene and hydrated using graded alcohol, following which antigen retrieval and blocking were performed. The sections were then incubated, first with primary antibodies against MeCP2 (Santa Cruz, CA, USA) or GIT1 (Santa Cruz) at a dilution of 1:200, and then with secondary antibodies. Measurement was performed using 3,3′-diaminobenzidine and hematoxylin. If >50% positive cells were observed in five random fields, the specimen was considered as showing high expressions of MeCP2 or GIT1.

### Luciferase reporter assay

The binding sites for miR-638 in the 3′-UTR of MeCP2 were synthesized (Beijing AuGCT DNA-SYN Biotechnology Co. Ltd, Beijing, China) and cloned into the pmirGLO vector (named MeCP2-WT), which also harbors firefly and *Renilla* reporter genes. The mutated 3′-UTR sequences of MeCP2 were also cloned and named MeCP2-MT (Supplementary Table S5). The sequences of constructed plasmids were confirmed by DNA sequencing (Sangon Biotech, Shanghai, China). The pre-miR-638 expression vector (Sangon Biotech) and WT or MT reporter plasmids were cotransfected into HEK293T cells. A dual-luciferase reporter assay was performed using the Dual-Luciferase Assay System (Promega, Madison, WI, USA) 24 h after transfection. Luciferase activity was normalized to Renilla luciferase activity. Experiments were performed at least three times.

### Plasmid construction and transient transfection

Human miR-638 precursor (pre-miR-638) was cloned into the pcDNA6.2-GW/EmGFP vector following the manufacturer’s instructions (Sangon Biotech). Full-length human MeCP2 complementary DNA was cloned into the pCMV2-GV146 vector (Genechem Co. Ltd). pCMV2-GV146-GFP-MeCP2 (WT), pCMV2-GV146-GFP-Mutation type 1 (MT1) and pCMV2-GV146-GFP-Mutation type 2 (MT2) plasmids were constructed (Supplementary Table S6). The reporter plasmid pGL3-GIT1 contained a 369-bp fragment spanning from 27916926 to 27917294 relative to the transcription start site of the GIT1 promoter, placed upstream of the firefly luciferase reporter gene (pGL3-GIT1-luc) (Genechem Co. Ltd). BGC-823 and AGS cells were cultured in medium without antibiotics for 24 h, and then the pcDNA6.2-GW/EmGFP vector, pre-miR-638 expression plasmid, pCMV2-GV146 vector or pCMV2-GV146-MeCP2 vector were transiently transfected into the cells using Lipofectamine 2000 (Invitrogen). After transfection, cells were cultured for 48 h to be used for assays.

### Lentiviral construction and cell transfection

Pre-miR-638-packaged lentiviruses (named LV-miR-638) were constructed by Genechem Co. Ltd to achieve miR-638 overexpression. Empty lentiviral vectors (named LV-Ctrl) were used as controls. MeCP2 shRNA lentiviral vectors (Genechem Company Ltd) were used to knockdown MeCP2 expression. The sequences are listed in Supplementary Table S5. The negative control lentiviral vector named sh-Ctrl. BGC-823 and AGS cells were cultured in a six-well plate and infected with 1 ml of viral stock for 10 h. The medium was then replaced using normal culture medium.

### siRNA/anti-miR-638 synthesis and transfection

siRNA was pre-designed for GIT1 gene silencing, and the sequences are listed in Supplementary Table S7. Small interfering RNA oligonucleotides were used as miR-638 inhibitors (named anti-miR-638; Supplementary Table S7). Scramble siRNA was used as a negative control (named anti-miR-Ctrl). The siRNAs and anti-miR-638 were synthesized by GenePharma Corporation (SGC, Shanghai, China). After cell culture for 24 h, GIT1 siRNAs or anti-miR-638 were transiently transfected into the BGC-823 and AGS cells using LipofectamineTM-2000 in accordance with the manufacturer’s protocol.

### Cell proliferation assay

Cell proliferation was assessed using MTT assays (Sigma, St Louis, MO, USA). BGC-823 and AGS cells were seeded at 5000 cells/well in 96-well plates. At the end of the culture period, 20 μl of MTT solution was added and cells were incubated for 4 h at 37 °C in a humidified incubator. Formazan crystals were dissolved in 150 μl of dimethyl sulfoxide. Absorbance was examined at a wavelength of 492 nm with a multi-microplate test system (POLARstar OPTIMA; BMG Labtechnologies, Offenburg, Germany).

### Colony formation assays

Cells were seeded at a density of 500 cells/well in 12-well plates 24 h after treatment and cultured for 14 days. The colonies were fixed, stained using 0.1% crystal violet, analyzed and normalized to the control group. Five parallel wells were used for each assay.

### Cell-cycle analysis

Cells were harvested at 48 h post-transfection, washed twice with phosphate-buffered saline, and fixed with 70% ice-cold ethanol at 4 °C overnight. After being washed again twice in phosphate-buffered saline, 0.1 mg/ml RNase A and 0.05 mg/ml propidium iodide was added and incubated for 15 min at room temperature. The distribution of cell-cycle stages was measured by flow cytometry (FACSCalibur, BD Biosciences, San Jose, CA, USA).

### Apoptosis analysis

Cells were harvested 48 h after transfection, washed twice with phosphate-buffered saline and then stained using an Annexin-V-FITC/propidium iodide Apoptosis Detection kit (Invitrogen). Flow cytometry was performed, and cell apoptosis was quantified by counting the number of stained cells.

### Tumorigenicity assays *in vivo*

All *in vivo* experiments were approved by the Institutional Committee of Animal Care and Use at Xi’an Jiaotong University. Five-week-old male BALB/C nude mice were used to investigate tumorigenicity. BGC-823 cells were infected with LV-Ctrl or LV-miR-638. The cells were resuspended in phosphate-buffered saline and were injected at a concentration of 1 × 10^6^ cells subcutaneously into both posterior flanks of the mice. Tumor growth was examined with a Vernier caliper every 3 days for a total of 28 days. Tumor volumes (*V*) were calculated by examining the length (*L*) and width (*W*) of tumors and using the formula: *V*=(*L* × *W*^2^)/2. *In vivo* bioluminescence imaging was performed using the Xenogen IVIS Spectrum imaging system (Xenogen, Alameda, CA, USA). Tumors were then removed and weighed. The tissues were embedded in paraffin for immunohistochemistry, and were frozen for qRT–PCR and western blot analysis.

### Western blot

The transfected GC cells, and grafted tumor cells, were lysed with RIPA buffer (Cell Signaling Technology, Boston, MA, USA). Equal amounts of protein lysates were run on 10% sodium dodecyl sulfate–polyacrylamide gel electrophoresis gels, and transferred to polyvinylidene fluoride membranes. The membranes were incubated with primary antibodies overnight at 4 °C, and were then incubated with corresponding secondary antibodies for 1 h. After that, the membranes were incubated with ECL (Pierce, Rockford, IL, USA) for chemiluminescence detection. The luminescent signal was detected and recorded using a Syngene GBox (Syngene, Cambridge, UK). The primary antibodies used are listed in Supplementary Table S8.

### Microarray analysis

BGC-823 cells were transfected with LV-Ctrl, LV-miR-638, anti-miR-Ctrl, anti-miR-638, shRNA-Ctrl or MeCP2 shRNA for 48 h, respectively. Total RNA was extracted using TRIzol Reagent and purified using the PureLink RNA mini kit (Invitrogen). Global gene expression was analyzed using the Human Gene Expression 4x44K v2 Microarray Kit (Agilent, Santa Clara, CA, USA). Total RNA extracted from BGC-823 cells was used for cDNA synthesis. The labeled cDNA was purified and hybridized to the microarray. Washing and staining of the arrays were performed according to the manufacturer’s instructions, and slides were scanned using an Agilent DNA Microarray Scanner (Agilent). Microarray experiments were performed by KangChen Biotech (KangChen, Shanghai, China). Data were obtained using the Agilent Feature Extraction software. Volcano plot filtering (fold change⩾2.0; *P*<0.05) was performed for evaluation of significant differential expression of mRNAs. Clustering was conducted based on the different expression levels of mRNAs using Cluster Treeview software from Stanford University (Stanford, CA, USA).

### ChIP, ChIP-Seq and ChIP–RT–PCR

BGC-823 cells were transfected with empty plasmid, pCMV2-GV146-GFP-MeCP2 plasmid (WT), pCMV2-GV146-GFP-Mutation type 1 plasmid (MT1) and pCMV2-GV146-GFP-Mutation type 2 plasmid (MT2) for ChIP. ChIP was performed as described previously.^53^ BGC-823 cells were crosslinked with 1% formaldehyde for 15 min and quenching was performed using glycine (125 mm). Nuclear lysates were sonicated using a cell cracker. The chromatin was sonicated into 200-bp (approx.) fragments. The lysates were divided into two portions and were incubated with 5 μg of antibodies against MeCP2, GFP or IgG (Supplementary Table S8) overnight at 4 °C. DNA-protein complexes were captured using Dynabeads Protein A (Invitrogen) and eluted in TE buffer at 65 °C. Crosslinking was then reversed for 8 h at 65 °C. Then, DNA was extracted using the QIAquick PCR purification kit (QIAGEN, Hilden, Germany). Sequencing was performed on an Illumina HiSeq 2000 using TruSeq Rapid SBS Kits (Illumina, San Diego, CA, USA, FC-402-4002). The locations of ChIP-enriched DNA present in the library were based on the Human Feb 2009 assembly and visualized using the genome browser of the University of California. Peak calling in the mapped ChIP-Seq data was performed with ChIP-Peak and subjected to further bioinformatics analysis. The ChIP-Seq experiments were performed by KangChen Biotech. In addition, analysis of DNA via RT–PCR was performed using gene-specific primers (Supplementary Table S9).

### GIT1-Luciferase reporter gene assay

BGC-823 cells were seeded into 96-well culture plates. Four wells were seeded per group. The cells were transfected with pGL3-luc, pGL3-GIT1-luc, or pGL3-GIT1-luc plasmids, sh-Ctrl or MeCP2 shRNA, MeCP2 overexpression vector or empty vector control, and were treated with methylation. Luciferase activity was measured at 48 h post-transfection using a Dual-Luciferase Reporter Assay System (Promega).

### Statistical analysis

All experiments were performed at least in triplicates unless otherwise stated. Significant between-group differences were estimated using Student’s *t*-test and Pearson’s *χ*^2^ test, as appropriate. Cell proliferation was analyzed using two-way ANOVA test. Relationships between miR-638 expression and clinicopathologic characteristics were analyzed using the *χ*^2^ test. Pearson’s correlation analysis was used to estimate the relationship between the GIT1 level and the levels of MeCP2 and miR-638. *P*-values<0.05 were considered to indicate statistical significance.
