# Supplementary figures and images for: MeCP2, a target of miR-638, facilitates gastric cancer cell proliferation through activation of the MEK1/2–ERK1/2 signaling pathway by upregulating GIT1
Source: Oncogenesis. 2017 Jul 31;6(7):e368–. doi: 10.1038/oncsis.2017.60 (PMC5541712; doi:10.1038/oncsis.2017.60)

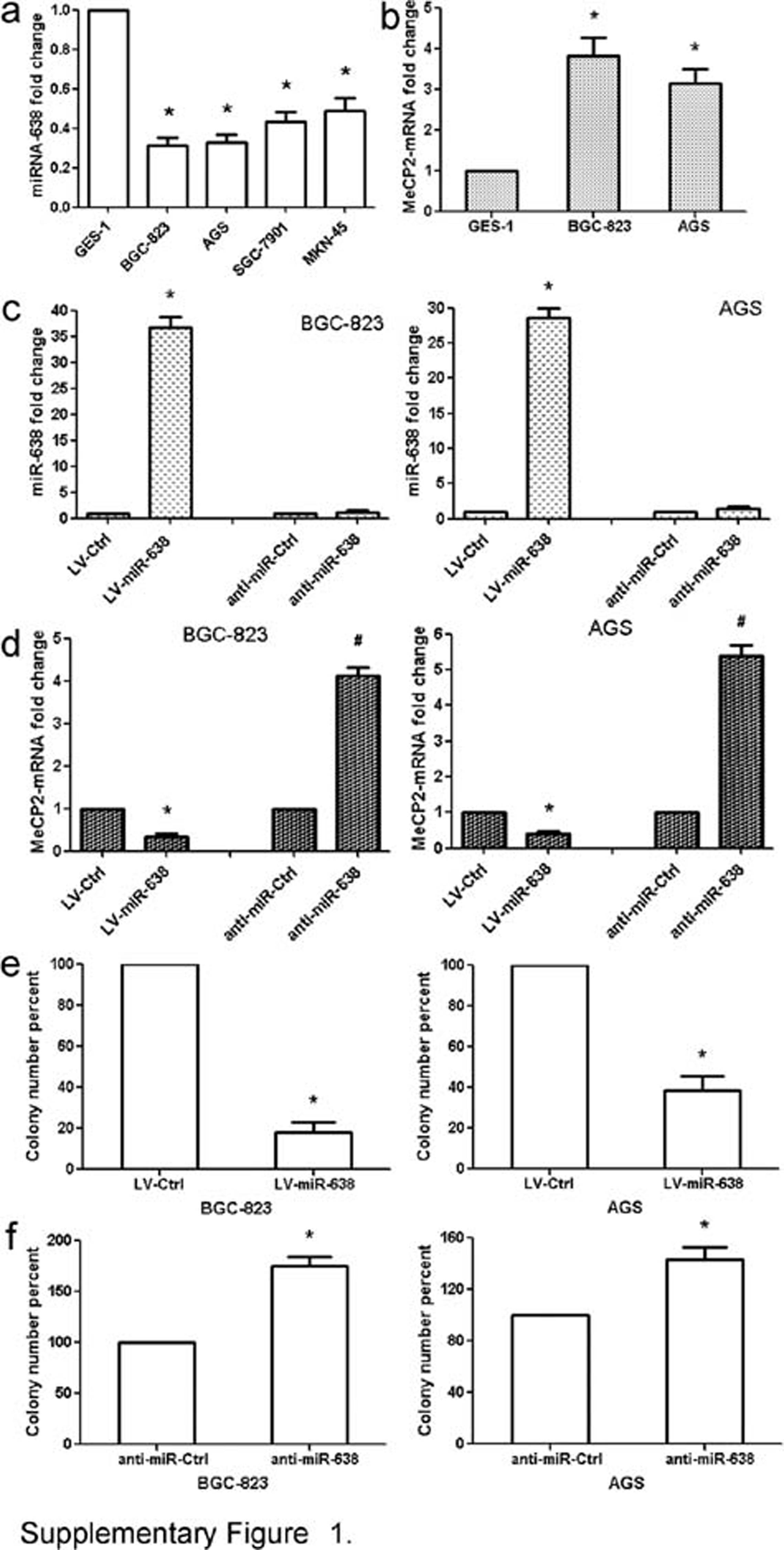

Supplement: Supplementary Figure 1 [file oncsis201760x1.tif]

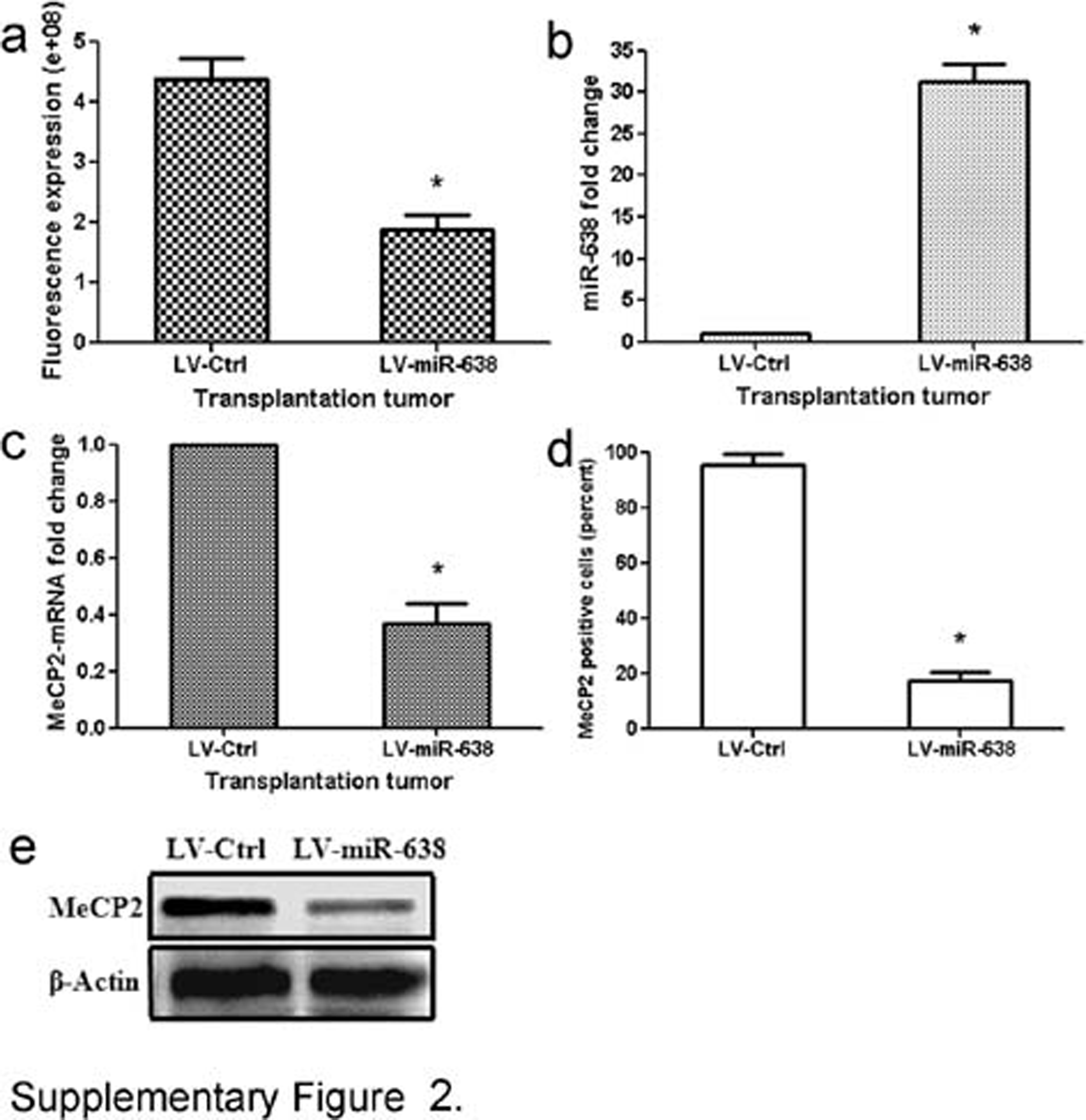

Supplement: Supplementary Figure 2 [file oncsis201760x2.tif]

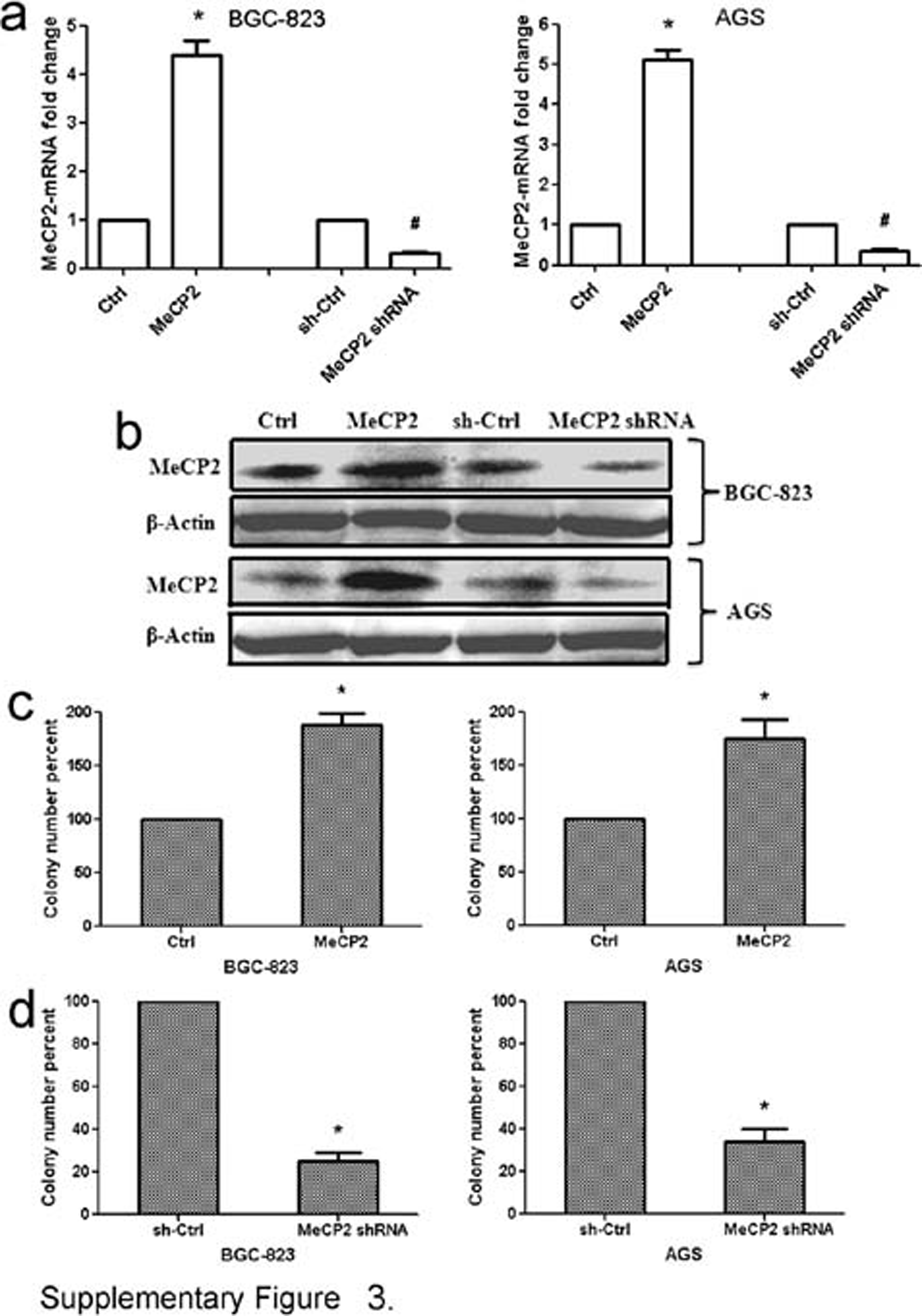

Supplement: Supplementary Figure 3 [file oncsis201760x3.tif]

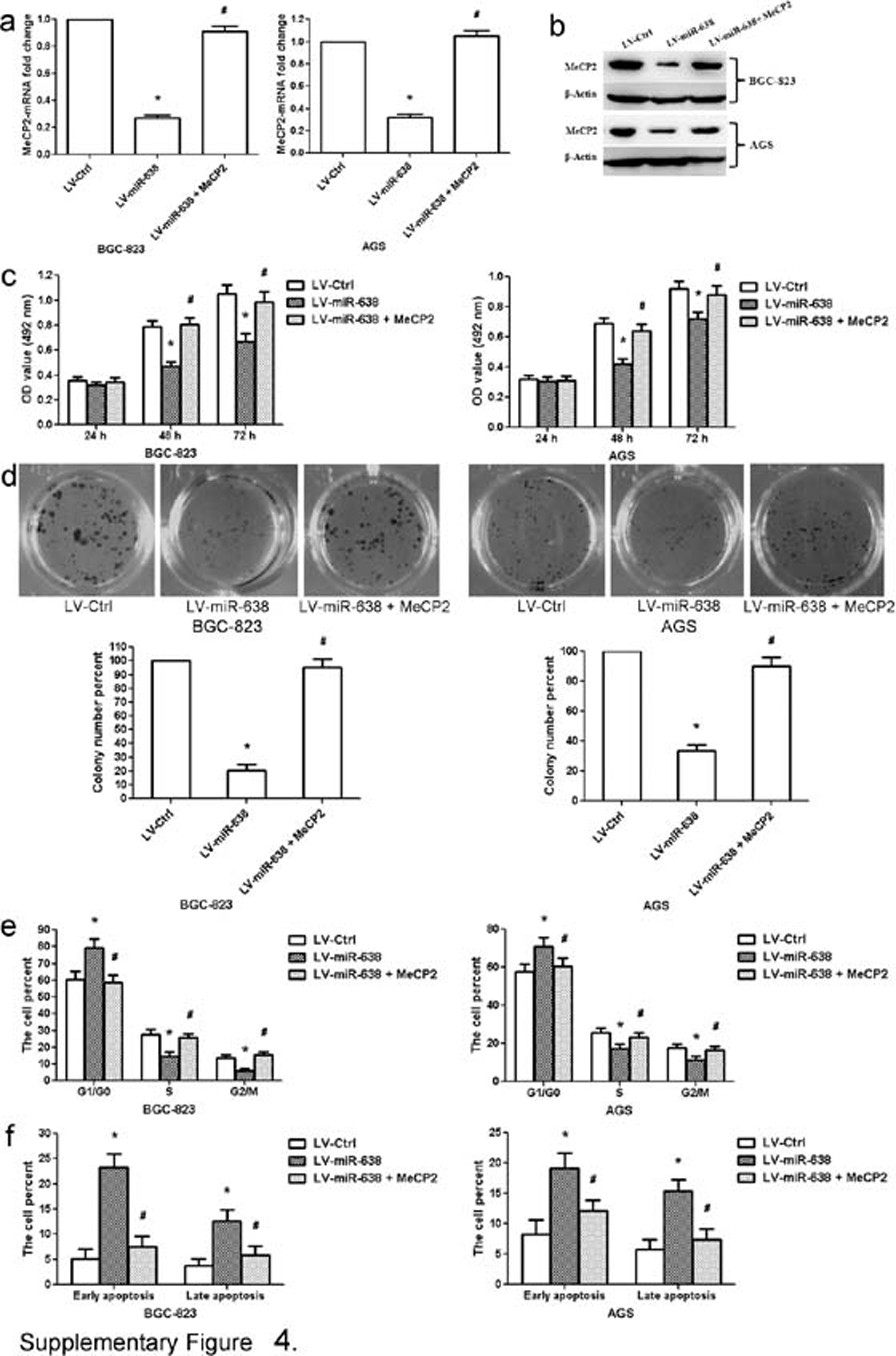

Supplement: Supplementary Figure 4 [file oncsis201760x4.tif]

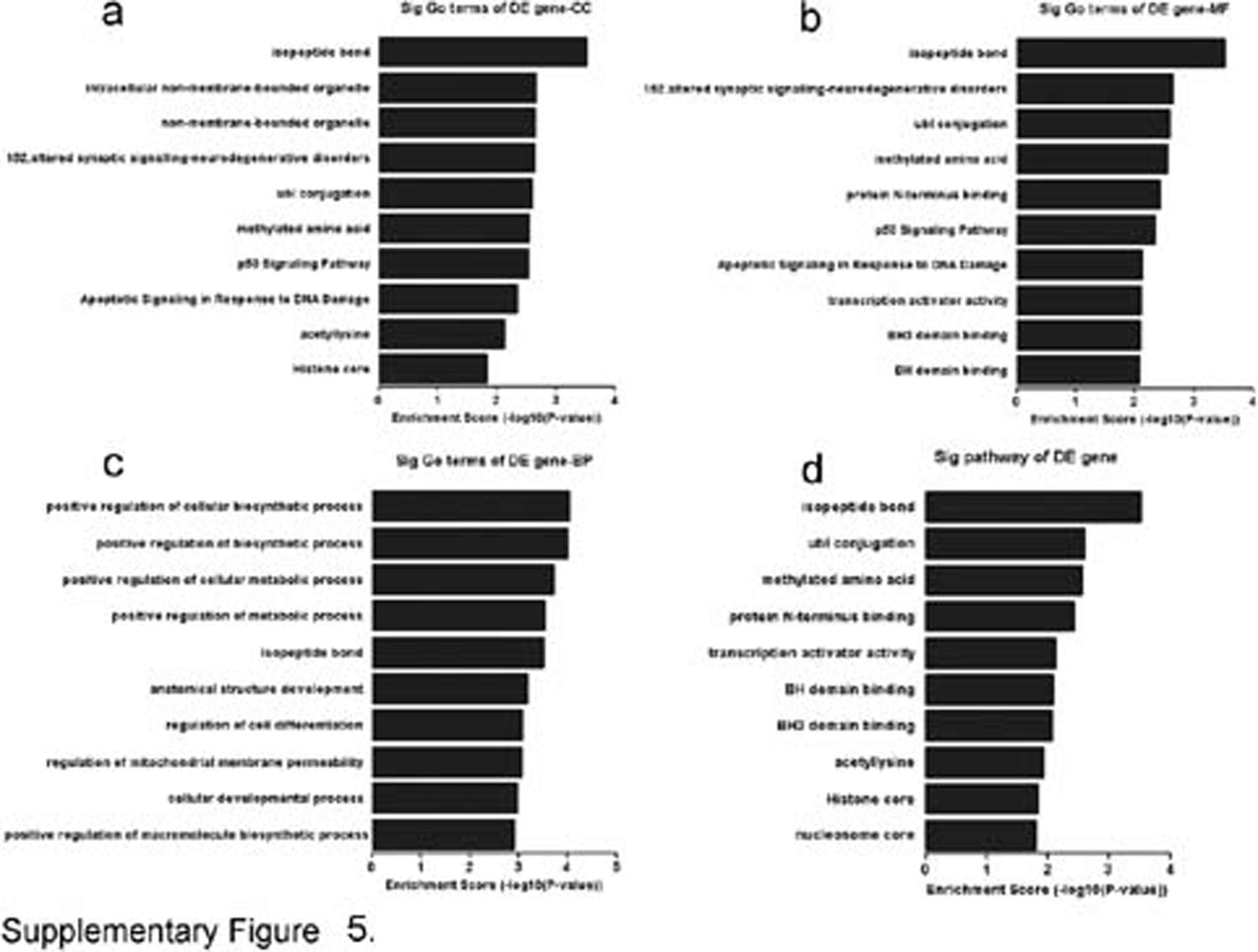

Supplement: Supplementary Figure 5 [file oncsis201760x5.tif]

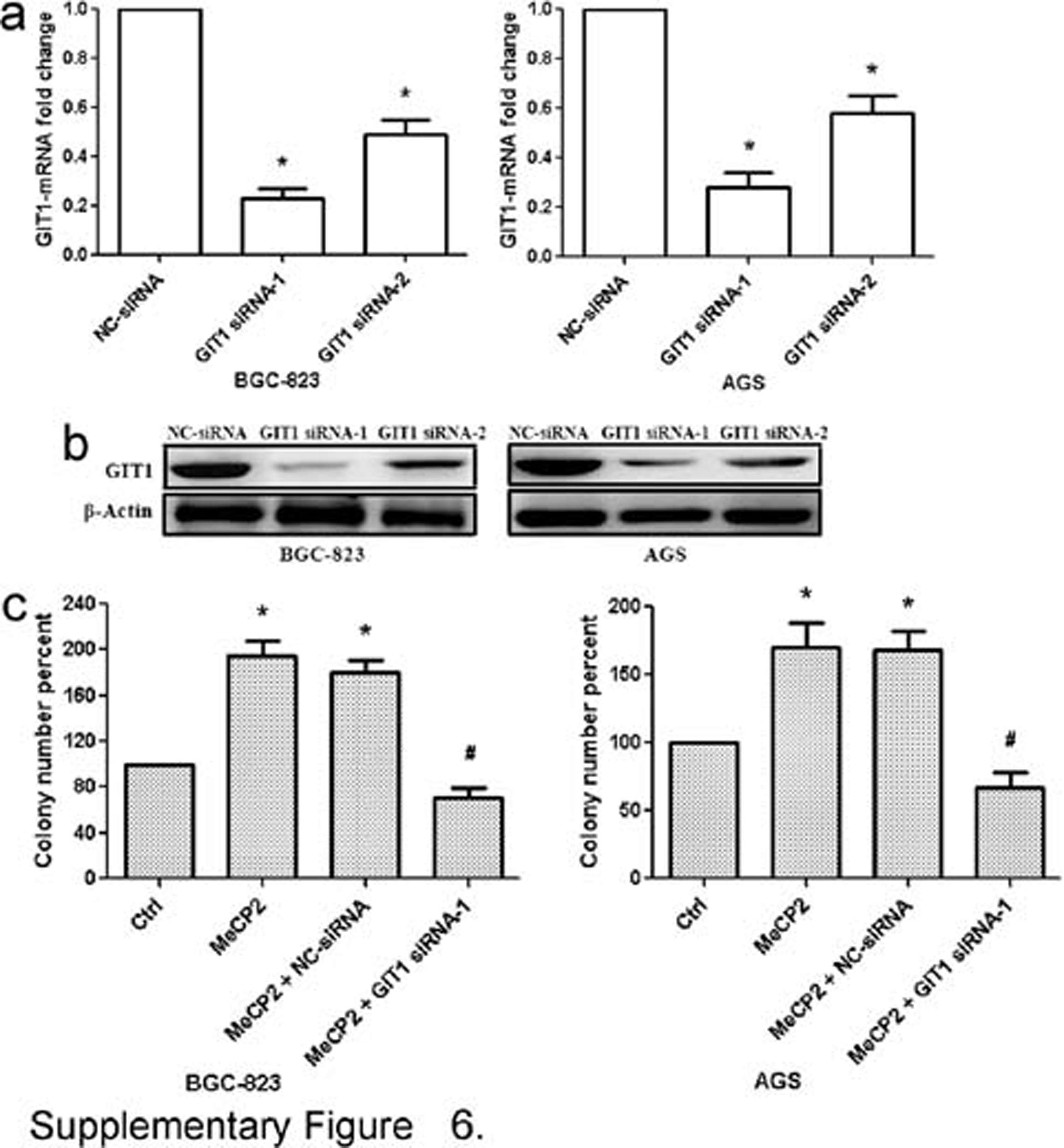

Supplement: Supplementary Figure 6 [file oncsis201760x6.tif]

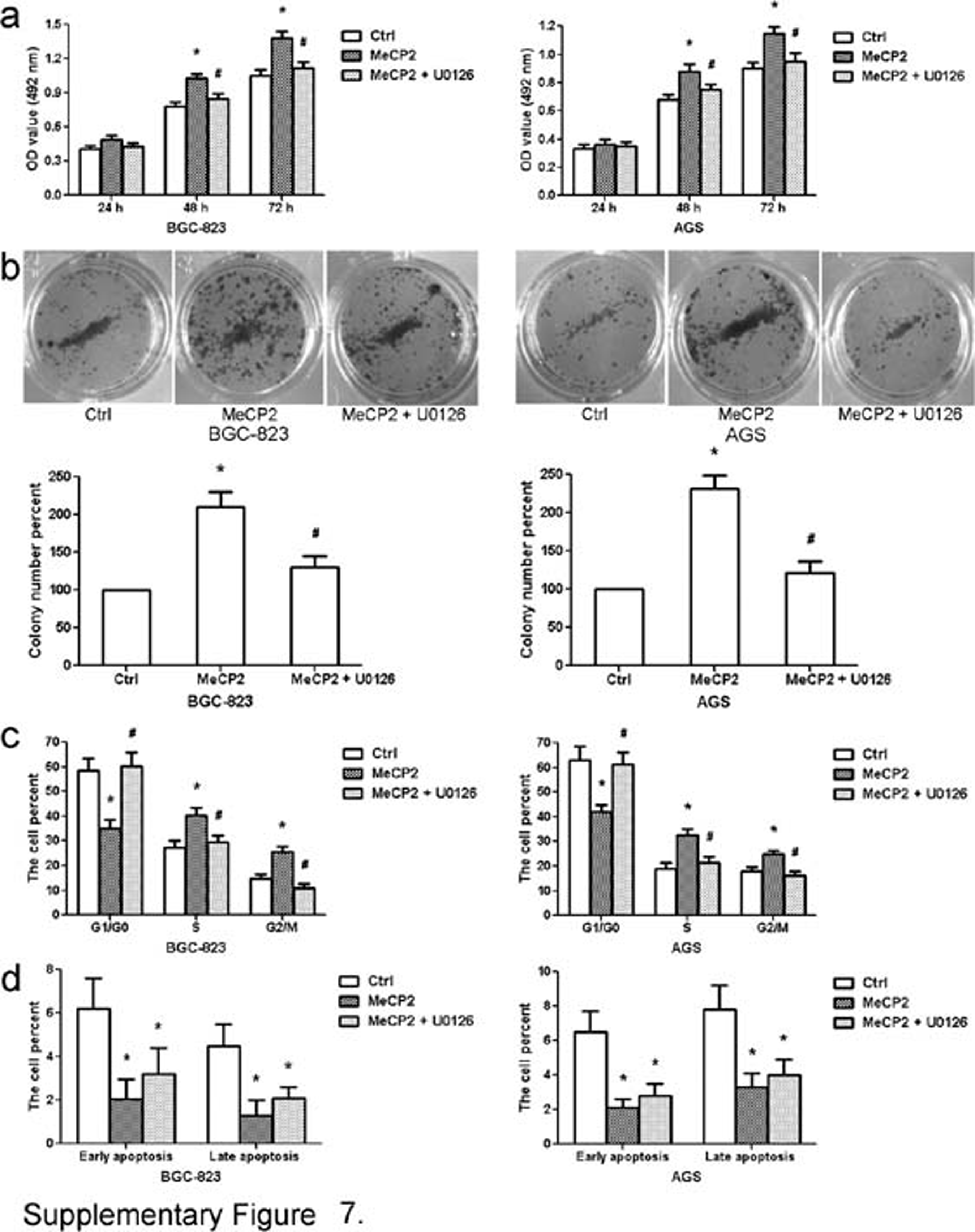

Supplement: Supplementary Figure 7 [file oncsis201760x7.tif]
