# Supplementary material for: MeCP2, a target of miR-638, facilitates gastric cancer cell proliferation through activation of the MEK1/2–ERK1/2 signaling pathway by upregulating GIT1
Source: Oncogenesis. 2017 Jul 31;6(7):e368–. doi: 10.1038/oncsis.2017.60 (PMC5541712; doi:10.1038/oncsis.2017.60)
Supplement: Supplementary Figure Legends [file oncsis201760x8.docx]

Supplementary Figure Legends

Supplementary Figure S1**.** miR-638 and MeCP2 mRNA expression, and colony number percent change in GC cells. (**a**) miR-638 expression in GC cell lines (BGC-823, AGS, SGC-7901, and MKN-45) and a human gastric epithelial cell line (GES-1). **P* < 0.01. (**b**) MeCP2 mRNA expression increased in GC cell lines (BGC-823 and AGS). **P* < 0.01. (**c**) Expression of miR-638 was evaluated by qRT-PCR after LV-miR-638 or anti-miR-638 (miR-638 inhibitor) treatment. (**d**) MeCP2 mRNA levels were examined by qRT-PCR. **P* < 0.01 compared with LV-Ctrl, #*P* < 0.01 compared with anti-miR-Ctrl, n = 3. (**e**) Colony number percent decreased after treated with LV-miR-638. **P* < 0.01. (**f**) Colony number percent increased after treated with anti-miR-638. **P* < 0.01.

Supplementary Figure S2. GC cell growth is inhibited after tumors are injected with LV-miR-638 cells. (**a**) At day 28, tumor growth was measured by bioluminescence imaging in vivo. **P* < 0.01, n = 5. (b) Levels of miR-638 expression in tumor xenografts were quantified by qRT-PCR. **P* < 0.01. (**c**) MeCP2 mRNA expression in tumor xenografts was examined by qRT-PCR. **P* < 0.01. (**d**) MeCP2 positive cell percent in tumor xenografts. **P* < 0.01. (**e**) MeCP2 protein expression in tumor xenografts was examined by western blot.

Supplementary Figure S3. MeCP2 expression levels and colony number percent change after transfection with MeCP2 shRNA and an MeCP2 overexpression vector. (**a**) MeCP2 mRNA expression was examined by qRT-PCR. **P* < 0.01, compared with Ctrl, #*P* < 0.01, compared with sh-Ctrl, n = 3. (**b**) MeCP2 protein expression was measured by western blot. (**c**) Colony number percent increased after treated with MeCP2 overexpression vector. **P* < 0.01. (**d**) Colony number percent decreased after treated with MeCP2 shRNA. **P* < 0.01.

Supplementary Figure S4. MeCP2 rescues miR-638–induced cellular phenotypes in GC cells. (**a**) Cells were cotransfected with MeCP2 overexpression vector and LV-miR-638 in BGC-823 and AGS cells. The expression level of MeCP2 was verified by qRT-PCR. (**b**) After cotransfected, MeCP2 expression was measured by Western blot analyses. (**c**) MTT assay was performed to determine the growth of GC cells after cotransfected with miR-638 and MeCP2. (**d**) Cell colonies were examined 12 days after cotransfection. (**e**) Cell cycle was determined 48 hours after cotransfection. (**f**) Apoptosis was measured 48 hours after cotransfection. **P* < 0.01 compared with LV-Ctrl, **#***P* < 0.01 compared with LV-miR-638. n = 3.

Supplementary Figure S5. GO functions of score enrichment terms in the overlapping genes with altered expression. (a) GO functions of score enrichment terms of CC. (**b**) GO functions of score enrichment terms of MF. (**c**) GO functions of score enrichment Terms of BP. (**d**) GO functions of score enrichment terms of pathways.

Supplementary Figure S6. Expression of GIT1 is silenced and colony number percent is decreased by siRNA. (a) GIT1 mRNA levels decreased in BGC-823 and AGS cells after transfection. **P* < 0.01, n = 3. (**b**) GIT1 protein levels were downregulated after transfection. (c) Colony number percent changed after treated with MeCP2 overexpression vector or MeCP2 + GIT1 siRNA-1. **P* < 0.01 compared with Ctrl cells, **#***P* < 0.01 compared with MeCP2 overexpression vector cells. n = 3.

Supplementary Figure S7. MEK1/2 inhibitor ( U0126) rescues MeCP2–induced cellular phenotypes in GC cells. (**a**) MTT assay was performed to determine the growth of GC cells after treated with MeCP2 overexpression vector and U0126 (15 μmol/L). (**b**) Cell colonies were examined 12 days after treated. (**e**) Cell cycle was determined 48 hours after treated. (**f**) Apoptosis was measured 48 hours after treated. **P* < 0.01 compared with Ctrl cells, **#***P* < 0.01 compared with MeCP2 overexpression vector cells. n = 3.
