# Supplementary material for: MeCP2, a target of miR-638, facilitates gastric cancer cell proliferation through activation of the MEK1/2–ERK1/2 signaling pathway by upregulating GIT1
Source: Oncogenesis. 2017 Jul 31;6(7):e368–. doi: 10.1038/oncsis.2017.60 (PMC5541712; doi:10.1038/oncsis.2017.60)
Supplement: Supplementary Tables [file oncsis201760x9.doc]

**Supplementary Table S1: Patient characteristics and clinicopathologic correlation of miR-638** expression

| Characteristics | Number of cases | | | miR-638 expression | | | P-value |
| --- | --- | --- | --- | --- | --- | --- | --- |
|  |  |  |  | High (n = 22) | | Low (n = 116) |  |
| Age | |  |  | |  | | 0.765 |
| ≥60 years | 61 | | 10 | | 51 | |  |
| <60 years | 77 | | 12 | | 65 | |  |
| Gender |  | |  | |  | | 0.432 |
| Male | 83 | | 14 | | 69 | |  |
| Female | 55 | | 8 | | 47 | |  |
| Histology |  | |  | |  | | 0.009 |
| Well | 57 | | 15 | | 42 | |  |
| Moderate | 38 | | 4 | | 34 | |  |
| poor | 43 | | 3 | | 40 | |  |
| Tumor size  <50 mm  ≥50 mm  Lymph node metastasis Yes  No  Lymphatic invasion  Yes  No  Venous invasion  Yes  No  T stage | 63  75  96  43  90  48  17  121 | | 14  8  6  16  15  7  2  20 | | 49  67  90  27  75  41  15  101 | | 0.015    0.026  0.825  0.916  0.653 |
| T1 | 51 | | 6 | | 45 | |  |
| T2 | 40 | | 5 | | 35 | |  |
| T3 | 31 | | 4 | | 27 | |  |
| T4 | 16 | | 7 | | 9 | |  |
| TNM Stage |  | |  | |  | | 0.762 |
| I | 38 | | 5 | | 33 | |  |
| II | 45 | | 8 | | 37 | |  |
| III | 32 | | 5 | | 27 | |  |
| IV | 23 | | 4 | | 19 | |  |

**Supplementary Table S2: ChIP-seq data of MeCP2 binding these 7 genes**

| Gene | Chr | Start position of peak | | End position of peak | Length | | Fold enrichment Strand | | |
| --- | --- | --- | --- | --- | --- | --- | --- | --- | --- |
| AR  GIT1  ALOX5  RASSF7  NFE2  UBC  DEFB103A | chrX  chr17  chr10  chr11  chr12  chr12  chr8 | 66765062  27916926  45871062  562593  54690342  125398278  7288875 | 66765363  27917294  45871377  562805  54690889  125398909  7289134 | | | 302  369  316  213  548  632  260 | | 36.92  25  25  25  20  18.33  17.5 | -  +  +  +  -  -  - |

**Supplementary Table S3. Patient characteristics and clinicopathologic correlation of GIT1** expression

| Characteristics | Number of cases | | | GIT1 protein expression | | | P-value |
| --- | --- | --- | --- | --- | --- | --- | --- |
|  |  |  |  | High (n = 60) | | Low (n = 16) |  |
| Age | |  |  | |  | | 0.941 |
| ≥60 years | 36 | | 29 | | 7 | |  |
| <60 years | 40 | | 31 | | 9 | |  |
| Gender |  | |  | |  | | 0.819 |
| Male | 45 | | 35 | | 10 | |  |
| Female | 31 | | 25 | | 6 | |  |
| Histology |  | |  | |  | | 0.008 |
| Well | 36 | | 23 | | 13 | |  |
| Moderate | 18 | | 17 | | 1 | |  |
| poor | 22 | | 20 | | 2 | |  |
| Tumor size  <50 mm  ≥50 mm  Lymph node metastasis Yes  No  Lymphatic invasion  Yes  No  Venous invasion  Yes  No  T stage | 33  43  59  17  51  25  9  67 | | 25  35  48  12  41  19  6  54 | | 8  8  11  5  10  6  3  13 | | 0.236    0.723  0.836  0.617  0.718 |
| T1 | 28 | | 22 | | 6 | |  |
| T2 | 21 | | 18 | | 3 | |  |
| T3 | 17 | | 12 | | 5 | |  |
| T4 | 10 | | 8 | | 2 | |  |
| TNM Stage |  | |  | |  | | 0.356 |
| I | 18 | | 14 | | 4 | |  |
| II | 25 | | 18 | | 7 | |  |
| III | 19 | | 16 | | 3 | |  |
| IV | 14 | | 12 | | 2 | |  |

**Supplementary Table S4: Primer sequence used for qRT-PCR in this study**

| Gene | Sequence |
| --- | --- |
| miR-638 reverse-transcribed | 5'-GTCGTATCCAGTGCGTGTCGTGGAGTCGGCAATTGCACTGGA  TACGACAGGCCGC-3' |
| U6 reverse-transcribed | 5'-CGCTTCACGAATTTGCGTGTCAT-3' |
| miR-638-F | 5'-ATCCAGTGCGTGTCGTG-3' |
| miR-638-R | 5'-TGCTAGGGATCGCGGGCGGGTG-3' |
| U6-F | 5'-GCTTCGGCAGCACATATACTAAAAT-3' |
| U6-R | 5'-CGCTTCACGAATTTGCGTGTCAT-3' |
| MeCP2-F | 5'-GCCGAGAGCTATGGACAGCA-3' |
| MeCP2-R | 5'-CCAACCTCAGACAGGTTTCCAG-3' |
| GIT1-F | 5'-CTAGCTAGCGCGTCGCCGCTGAGGA-3' |
| GIT1-R | 5'-GGAATTCGGGGCGCATGTACGGA-3' |
| β-Actin-F | 5'-TGGCACC CAGCACAATGAA-3' |
| β-Actin-R | 5'-CTAAGTCATAGTCCGCCTAGAAGCA-3' |

**Supplementary Table S5:** Sequences of recombinant plasmids

| Name | Sequence | |
| --- | --- | --- |
| MeCP2-WT Sense Strand | 5'- CCAGTGGCTATGGCCTGTGCGATCCCAC-3' |  |
| MeCP2-WT Antisense Strand | 5'-TCGATGGGATCGCACAGGCCATAGCCACTGAGCT-3' | |
| MeCP2-MT Sense Strand | 5'- CAGTGGCTATGGCCTGTGCGACGGCAC-3' | |
| MeCP2-MT Antisense Strand | 5'-TCGATGCCGTCGCACAGGCCATAGCCACTGAGCT-3' | |
| miR-638 Sence | 5'-AATCGTGAGCGGGCGCGGCAGGGATCGCGGGCGGGTGGCGGCCTAGGGCGCGGAGGGCGGACCGGGAATGGCGCGCCGTGCGCCGCCGGCGTAACTGCGGCGCTA-3' | |
| miR-638 Anti-sence | 5'-AGCTTAGCGCCGCAGTTACGCCGGCGGCGCACGGCGCGCCATTCCCGGTCCGCCCACCGCGCCCTAGGCCGCCACCCGCCCGCGATCCCTGCCGCGCCCGCTCACG-3' | |
| shRNA Negative Control  MeCP2 shRNA | 5′-AAAAGAGGCTTGCACAGTGCATTCAAGACGTGCACTGTGCAAGCCTCTTTT-3′  5'-TGCTTAAGCAAAGGAAATCTCTCGAGAGATTTCCTTTGCTTAAGCTTTTTTC-3' | |

**Supplementary Table S6: Sequences of MeCP2 MT** recombinant plasmids

| Name | Sequence |
| --- | --- |
| MeCP2-WT MBD sequence | 5'-AGGGGCCCTATGTATGATGACCCTACACTGCCCGAGGGCTGGACCAGGAAACTGAAGCAGAGGAAGTCCGGA**AGGAGCGCCGGCAAATACGAT**GTCTACCTGATTAACCCCCAGGGCAAGGCCTTTAGATCCAAGGTGGAGCTG**ATCGCCTACTTTGAGAAGGTC**GGCGACACATCCCTAGACCCGAATGACTTCGACTTCACAGTGACCGGCAGAGGA-3' |
| MeCP2-MT1 MBD sequence | 5'-AGGGGCCCTATGTATGATGACCCTACACTGCCCGAGGGCTGGACCAGGAAACTGAAGCAGAGGAAGTCCGGA**GATCAGTATCACGAAGGTCGC**GTCTACCTGATTAACCCCCAGGGCAAGGCCTTTAGATCCAAGGTGGAGCTGATCGCCTACTTTGAGAAGGTCGGCGACACATCCCTAGACCCGAATGACTTCGACTTCACAGTGACCGGCAGAGGA-3' |
| MeCP2-MT2 MBD sequence | 5'-AGGGGCCCTATGTATGATGACCCTACACTGCCCGAGGGCTGGACCAGGAAACTGAAGCAGAGGAAGTCCGGAAGGAGCGCCGGCAAATACGATGTCTACCTGATTAACCCCCAGGGCAAGGCCTTTAGATCCAAGGTGGAGCTG**CATGACAAGCAGGCCGAGAGT**GGCGACACATCCCTAGACCCGAATGACTTCGACTTCACAGTGACCGGCAGAGGA-3' |

**Supplementary Table S7:** Sequences of siRNA and anti-miR-638

| Name | Sequence | |
| --- | --- | --- |
| negative siRNA (NC-siRNA) sense | 5′-UUCUCCGAACGUGUCACGUTT-3′ |  |
| negative siRNA (NC-siRNA) antisense | 5′- ACGUGACACGUUCGGAGAATT-3′ | |
| GIT1 siRNA-1 sense | 5′-CCUUGAUCAUCGACAUUCUTT-3′ | |
| GIT1 siRNA-1 antisense | 5′-AGAAUGUCGAUGAUCAAGGTT-3′ | |
| GIT1 siRNA-2 sense | 5'-CGAGCUGCUUGUAGUGUAUTT-3' | |
| GIT1 siRNA-2 antisense | 5'-AUACACUACAAGCAGCUCGTT-3' | |
| anti-miR-Ctrl | 5'-CAGUACUUUUGUGUAGUACAA-3' | |
| anti-miR-638 | 5'-AGGCCGCCACCCGCCCGCGAUCCCU-3' | |

**Supplementary Table S8: Information on antibodies used for the correlation analysis**

| Antibody | WB | IHC | Specificity | | Company | |
| --- | --- | --- | --- | --- | --- | --- |
| MeCP2 (sc-20700)  GIT1 (sc-365084)  Phospho-MEK1/2 (2338)  MEK1/2 (4694)  Phospho-ERK1/2 (4377)  ERK1/2 (4695)  Phospho-c-Jun (3270)  c-Jun (9165)  Phospho-c-Fos (5348)  c-Fos (2250)  Cyclin D1 (2978)  β-Actin (sc-8432)  MeCP2 ChIP Grade (ab2828)  GFP ChIP Grade (ab290)  IgG ChIP Grade (ab171870) | 1:1000  1:1000  1:1000  1:1000  1:1000  1:1000  1:1000  1:1000  1:1000  1:1000  1:1000  1:3000  -  -  - | 1:200  1:200  -  -  -  -  -  -  -  -  -  -  -  -  - | Rabbit polyclonal  Mouse Monoclonal  Rabbit Monoclonal  Mouse Monoclonal  Rabbit Monoclonal  Rabbit Monoclonal  Rabbit Monoclonal  Rabbit Monoclonal  Rabbit Monoclonal  Rabbit Monoclonal  Rabbit Monoclonal  Mouse Monoclonal  Rabbit polyclonal  Rabbit polyclonal  Rabbit polyclonal | Santa Cruz Biotechnology  Santa Cruz Biotechnology  Cell Signaling Technology  Cell Signaling Technology  Cell Signaling Technology Cell Signaling Technology  Cell Signaling Technology  Cell Signaling Technology  Cell Signaling Technology  Cell Signaling Technology  Cell Signaling Technology  Santa Cruz Biotechnology  Abcam  Abcam  Abcam | |  |

**Supplementary Table S9: Primer sequence used for ChIP-qRT-PCR**

| Gene | Sequence |
| --- | --- |
| GIT1-F | 5'-TATCTCTGGTTGGGGTGAGGTGTGC-3' |
| GIT1-R | 5'-CCTGACCTTTCTGGCACCTGGGCGT-3' |
| GAPDH-F | 5'-GTGGCAAAGTGGAGATTGTT-3' |
| GAPDH-R | 5'-CTCGCTCCTGGAAGATGG-3' |
